# Porous Refractories Synthesized Using Rice Husk and Rice Husk Processing Products

**DOI:** 10.3390/ma18215063

**Published:** 2025-11-06

**Authors:** Svetlana Yefremova, Sergey Yermishin, Askhat Kablanbekov, Baimakhan Satbaev, Nurgali Shalabaev, Serik Satbaev

**Affiliations:** 1 RSE National Center on Complex Processing of Mineral Raw Materials of the Republic of Kazakhstan, Almaty 050036, Kazakhstan; esv-ret@mail.ru (S.Y.); kablanbekov_as@mail.ru (A.K.); 2 RSE Astana Branch of the National Center on Complex Processing of Mineral Raw Materials of the Republic of Kazakhstan, Nur-Sultan 010000, Kazakhstan; fnc-astana@mail.ru (B.S.); nurgali_s@bk.ru (N.S.); ssatbayev@gmail.com (S.S.)

**Keywords:** rice husk, high-temperature materials, porous refractories, lightweight refractories, thermal insulating materials

## Abstract

In recent years, research in the field of the sustainable production of refractory ceramics has become topical. Significant attention has been paid to the use of secondary raw materials for obtaining high-quality materials. The purpose of the current study was to develop new high-temperature porous materials based on the magnesium sulfate-refractory clay–chamotte–aluminum system using environmentally friendly raw components. To synthesize porous refractories, rice husk and the by-products of its thermal processing were used as substitutes for ingredients usually introduced into the composition of high-temperature materials. Ground rice husk was used as both a burnout additive and a silica source. It was added to the mixture instead of chamotte. An organic condensate from rice husk pyrolysis was used as a binder. A sodium silicate solution, after activating pyrolyzed rice husk with alkali, was also tested as a binder. These liquid ingredients served as replacements for lignosulfonate and liquid glass. The new raw material components and the porous refractories obtained with their use were studied using methods of chemical analysis, XRD, GC-MS, TA, SEM, and EDS. Standard methods for studying the properties of refractories were used to evaluate the physicomechanical and thermal characteristics of the experimental materials. The sample with the maximum content of rice husk (14.4 wt.%) and organic condensate from its pyrolysis (10.5 wt.%) demonstrated promising properties as a light porous refractory: an apparent porosity of 44%, a volumetric weight of 1.1 g·cm^−3^, compressive strength of 2.1 MPa, tensile strength in bending of 4.5 MPa, bond strength of 0.01 MPa, thermal shock resistance of 155 thermal cycles, and thermal conductivity of 0.05 W (m·K)^−1^. It can be used as a prospective thermal insulating material.

## 1. Introduction

The Republic of Kazakhstan is the ninth-largest country in the world in terms of territorial area (2724.9 thousand km^2^). More than half of the land is agricultural land. In this regard, agriculture is one of the most important sectors of the economy. In the agro-industrial complex, a developed sphere of activity is the production of grain, which is supplied to dozens of countries. The list of main cultivated crops is dominated by wheat, barley, cotton, and rice.

On the other hand, Kazakhstan is rich in mineral resources such as copper, iron, aluminum, lead, natural gas, and oil. The mining and metallurgical industry is a leading sector in the industrial sector due to its rich raw material base. However, the operation of large metallurgical enterprises is associated with high consumption of energy, heat emissions, and waste generation [1,2,3]. Solving specific production issues is a direct task for domestic researchers. For instance, a scientific direction of creating new refractory materials based on the technology of self-propagating high-temperature synthesis (SHS) has been established [4]. The developed SHS technology for refractory production provides a significant reduction in the firing temperature of products and the time required for their manufacture compared to traditional methods. Traditionally, the production of refractory materials occurs at high (around 1800–1850 °C) firing temperatures. A charge of a certain composition is prepared when using SHS technology. When the charge is heated to a temperature of 850–900 °C, an exothermic reaction occurs. In the course of this reaction, the components of the refractory materials are sintered into a monolith. This allows the lining of metallurgical furnaces to operate at high temperatures (around 1900–2000 °C). The advantage of SHS technology is the synthesis of the refractory as a result of redox reactions from the combustion of aluminum with both oxidizers and a binder [5].

A common system for producing refractories using SHS technology is Al–chamotte–MgSO_4_. By varying the contents of the ingredients, it is possible to influence the compressive strength and porosity of the resulting refractories. Due to their considerable cost, it seems expedient to test the use of burnout additives for producing porous high-temperature materials. The testing of the Al–chamotte–MgSO_4_ system with burnout additives of plant origin has not been previously performed.

The literature provides examples of testing wood sawdust and rice husk (RH) as burnout additives in refractory production [6,7]. Rice husk is a multi-tonnage waste generated during rice production. It contains lignin, carbohydrates (cellulose and hemicellulose), nitrogenous substances, vitamins, organic acids, and mineral components. The predominant mineral component of rice husk is silicon dioxide. The quantitative contents of the constituent components of rice husk depend on various factors (rice variety, cultivation conditions, method for determining the components, etc.) and, according to various researchers, varies over a wide range. For example, the ash content ranges from 13 to over 29% by mass. [8]. The main share (80–95% by mass) in the ash of rice husk is silicon dioxide [9,10]. Novembre et al. [11] managed to obtain ash with a content of almost 99% silicon dioxide. However, obtaining rice husk ash with such a high content of silicon dioxide is associated with high energy costs (calcination at 550 °C for 6 h) and environmental pollution by flue gases.

Rice husk accumulates in large quantities as it constitutes on average more than 20% of the mass of unhusked rice. Due to its high content of silicon dioxide, RH is not subject to decay and creates environmental problems. That is why for many decades, rice husk has been the object of research by scientists in search of environmentally friendly methods for its processing. The predominant direction of research is the use of RH as a raw material for the production of sorbents [12]. Works related to the use of rice husk ash as a source of silicon dioxide dominate the studies on the creation of refractory materials [13,14,15].

In scientific research, the study of the method for extracting silicon dioxide from the composition of rice husk is of independent interest [16]. Various options for silicon dioxide production have been proposed in the literature. One of the most common is the thermal treatment of rice husk under various conditions [9,17,18]. Chemical pretreatment of the raw material is also practiced to increase the purity of the obtained ash [19,20]. The most chemically pure silicon dioxide is obtained as a result of treating the carbonizate of rice husk with an alkaline solution followed by precipitation with acid. It has been shown [6,13,21] that silica from rice husk can be successfully used in the production of construction and high-temperature materials. The use of silica from rice husk is equally favorable for obtaining both porous and high-strength [13,14] types of refractories.

To create lightweight porous refractories, Thang et al. [6] used rice husk ash (RHA) instead of diatomite (DE) in equal proportions (at 32.5–50 wt.%). Experimental samples are characterized by high stability (thermal shock resistance times of 61–78) and low thermal conductivity (up to 0.1 W (m·K)^−1^), confirming their compliance with the requirements of modern standards.

Sobrosa et al. [22] obtained refractory ceramics by replacing part of kaolin (KC, wt.%: SiO_2_—57.83; CaO—0.13; MgO—0.36; Fe_2_O_3_—2.25; Al_2_O_3_—27.52; Na_2_O—<0.001; K_2_O—1.87; TiO_2_—0.38; MnO—<0.01; loss to fire—8.63) with rice husk silica (RHS, wt.%: SiO_2_—91.48; CaO—0.36; MgO—0.32; Fe_2_O_3_—0.05; Al_2_O_3_—ND; Na_2_O—0.04; K_2_O—1.40; TiO_2_—0.003; MnO—0.32; SO_3_—0.15; P_2_O_5_—0.45; loss to fire—3.50). It was found that rice husk silica promotes denser packing of the charge, which improves the mechanical properties of the experimental samples. With the introduction of 20 vol.% RHS, compressive strength increased by 20% relative to the control sample, reaching 140 MPa. However, only one sample, containing no more than 10 vol.% RHS, passed the test for thermal shock resistance and was recommended for use.

Stochero et al. [23] also replaced kaolin (KC, wt.%: SiO_2_—57.83; CaO—0.13; MgO—0.36; Fe_2_O_3_—2.25; Al_2_O_3_—27.52; Na_2_O—<0.001; K_2_O—1.87; TiO_2_—0.38; MnO—<0.01; loss to fire—8.63) with silica from rice husk (RHS, wt.%: SiO_2_—91.48; CaO—0.36; MgO—0.32; Fe_2_O_3_—0.05; Al_2_O_3_—ND; Na_2_O—0.04; K_2_O—1.40; TiO_2_—0.003; MnO—0.32; SO3—0.15; P_2_O_5_—0.45; loss to fire—3.50). However, steel fibers were added in order to improve the indicators of thermal properties, in particular, thermal shock resistance. The use of a mixture of kaolin, rice husk ash, and steel fibers in the ratio (vol.%) 71:20:9 allowed for the production of refractory ceramics with acceptable mechanical (tensile strength in bending—13 MPa) and thermal (average resistance to thermal shock—0.36) properties.

Silva et al. [24] studied the replacement of a part (10 wt.%) of the refractory clay (A, wt.%: SiO_2_—23.19; Al_2_O_3_—54.40; Fe_2_O_3_—4.98; other oxides—17.43) with rice husk ash (S, wt.%: SiO_2_—89.06; other oxides—10.94) and its combination with wollastonite microfibers (W, wt.%: SiO_2_—43.25; CaO—55.19; other oxides—1.59). They observed that the introduction of wollastonite up to 20 wt.% worsens the mechanical (linear retraction after the sintering process drops from 7.78 to 1.64%) and thermal (thermal shock resistance at 500 °C decreases from 4 to 1 cycle) properties of refractories because of the formation of high porosity (25% versus 16% for the sample without W but containing 10% S; versus 13% for the sample without S and W).

Hossain et al. [25] produced insulating bricks using ground and unground rice husk ash RHA (aggregates) up to 50% by mass, isolated via alkaline extraction sol (binder) from 2.5 to 12.5% by mass with sodium hexametaphosphate (SHMP) added in small amounts (0.2% by mass). They found that the bricks, obtained by firing at 1000 °C, are characterized by a porous structure (apparent porosity 67%). The bricks meet the requirements for insulation and can be used in various furnaces. In the case of firing at higher temperatures, the degree of structure ordering increases due to the appearance of crystalline phases. This negatively affects the thermal properties of ceramic materials.

Gonçalves et al. [9] obtained thermal insulators based on rice husk ash (RHA) via extrusion (Ex) and pressing (Pr) methods instead of industrial diatomaceous silica (DS) and noted a dependence between the degree of ordering of the ceramics and their thermal properties. They showed that RHA Ex, RHA Pr, and DS samples had similar physical and mechanical characteristics. However, their thermal properties varied depending on the ordering of the structure. The higher the degree of structure disorder, the lower thermal conductivity of thermal insulators. As a material with a very disordered structure, DS has better thermal conductivity values. Despite the higher thermal conductivity values of RHA Ex (0.24 W (m·K)^−1^) and RHA Pr (0.2 W (m·K)^−1^) samples than DS (0.16 W (m·K)^−1^), they are generally small and, along with their high porosity (50–75%) and low density (0.36–0.73 g·cm^−3^), allow for the use of rice husk ash as a feedstock for the production of thermal insulators.

To produce high strength refractory materials, Bhardwaj et al. [26] replaced mass quartz with rice husk ash (RHA) from 20 to 100% in a mixture with clay and refractory grog. RHA was obtained by burning rice husks at 500 °C with additional firing at 600 °C for 2 h. The resulting material had the following composition (wt.%): SiO_2_—92.81; Na_2_O—2.658; P_2_O_5_—1.071; K_2_O—1.021; CaO—0.417; Fe_2_O_3_—0.312; MgO—0.212; RuO_2_—0.151; SO_3_—0.132; TiO_2_—0.112; ZnO—0.091; CuO—0.058; Rb_2_O—0.036; BaO—0.031; ZrO_2_—0.025; Re_2_O_7_—0.021; Y_2_O_3_—0.012; Eu_2_O_3_—0.010. The experimental refractory samples had good physical, chemical, and thermal properties. The quartz-based material and RHA-based refractory showed maximum refractoriness of 1580 °C and 1470° C, respectively. A composition with a replacement of 60% of quartz with RHA was recommended as an optimal composition.

Sembiring et al. [27,28] fabricated cordierite from rice husk silica. Silica was obtained via treatment of rice husk with alkali under boiling conditions followed by precipitation via the action of hydrochloric acid. Silica was mixed with magnesium and aluminum in a ratio of 5:2:2 by mass. The charge was sintered at temperatures from 1050 to 1350 °C. The cordierite phase was predominant at a sintering temperature of 1230–1350 °C. Aluminum–cordierite samples with different mass ratios of cordierite to aluminum oxide (100:0, 95:5, 90:10, 85:15, 80:20, 75:25, 70:30) were obtained from these materials. Aluminum-enriched (30% Al_2_O_3_) refractory cordierite is mainly composed of spinel, corundum, and cristobalite. It has high values of density (3.7 g·cm^−3^), hardness (23 GPa), mechanical (bending strength—140 MPa), and thermal properties (coefficient of thermal expansion—9.5 × 10^−6^ (°C)^−1^) at low porosity (3%). Rice husk silica-based refractory cordierite samples have the properties of a promising insulating material, while aluminum-enriched dense samples could be used as abrasive materials.

To produce mullite ceramics, Serra et al. [29] used ash from the industrial combustion of rice husk. It was mixed with α-Al_2_O_3_ in a stoichiometric ratio of 3Al_2_O_3_/2SiO_2_. The ingredients were milled in a ball mill, pressed, and fired at temperatures from 1100 to 1600 °C. It was determined that the mullitization reaction begins at 1400 °C and ends at 1600 °C, and it was shown that produced mullite ceramics could be used to prepare different refractories. For example, to produce porous refractories, it is necessary to increase the porosity of the final product by introducing combustible additives for its use as insulating materials. Wood sawdust is used as a burnout additive during the production of porous high-temperature materials using silicon dioxide from rice husk [6]. However, from the standpoint of creating sustainable and cost-effective materials, it is highly relevant to study the possibility of using rice husk as both a burnout additive and a silica-containing raw material. Such studies are few [7].

Arthur and Gikunoo [7] studied the properties of thermal insulation materials made from Ghanaian red anthill clay (RAC) enhanced with sawdust (SD), rice husk (RH), and their mixture. It was shown that mechanical (compressive strength—0.981–6.867 MPa) and thermal (0.01–1.1 W (m·K)^−1^) properties corresponded to the standard values for refractory bricks. At the same time, the required (20 cycles) value of thermal shock resistance was achieved by samples with vegetable additive content above 15%. Thus, the possibility of using rice husk to obtain porous high-temperature materials has been demonstrated.

Since, as mentioned above, Kazakhstan is a rice-cultivating country, the problem of processing rice husk is also relevant for our country [30]. This year, our enterprise has launched a facility for the thermal processing of rice husk to produce a silica–carbon composite (SCC). The installation’s capacity is 1500 t of silica–carbon composite per year. The process is based on the pyrolysis of rice husk in a rotating reactor in an atmosphere of flue gases without air access. The rotation of the reactor ensures mixing and uniform heating of the rice husk. The plant-based raw material is placed in the heating zone for 30 min, and the pyrolysis temperature is regulated from 350 to 650 °C. This temperature is set according to the requirements for the composition of the silica–carbon composite depending on its further use. The main components of the silica–carbon composite are carbon (49–55 wt.%) and silicon dioxide (35–40 wt.%), present in an amorphous form. Due to the removal of flue gases, the pressure in the reactor is maintained at 1 atm. The installation is equipped with a condensation system to capture the flue gases in order to reduce the environmental impact. Accordingly, an organic condensate (OC-RH) is formed as a by-product.

One of the applications for the silica carbon composite is the production of a carbon sorbent. The process of obtaining the carbon sorbent consists of activating SCC with water steam at 850 °C followed by treatment with a sodium hydroxide solution. This leads to the formation of a sodium silicate solution (SS-RH) as a by-product.

In this context, the present study is aimed at creating porous high-temperature materials based on the magnesium sulfate-refractory clay–chamotte–aluminum system with the replacement of chamotte by rice husk and commercial binders (lignosulfonate and liquid glass) with rice husk processing products (OC-RH and/or SS-RH) to ensure maximum utilization of the multi-tonnage waste and by-products of its processing.

The synthesis of new porous high-temperature materials was investigated using a range of physicochemical analysis methods, including X-ray diffraction (XRD) analysis, thermal analysis (TA), scanning electron microscopy (SEM), and energy-dispersive spectroscopy (EDS), to study the ongoing processes. The physical and mechanical properties (apparent porosity, density, volumetric weight, compressive strength, bond strength, and tensile strength in bending) as well as thermal characteristics (thermal conductivity, deformation temperature under load, and thermal shock resistance) were investigated in accordance with the technical regulations currently in force in Kazakhstan. Correlation analysis and comparative evaluation with the properties of refractory materials reported in the literature were also performed.

## 2. Materials and Methods

### 2.1. Materials and Mix Design

**Figure 1:**
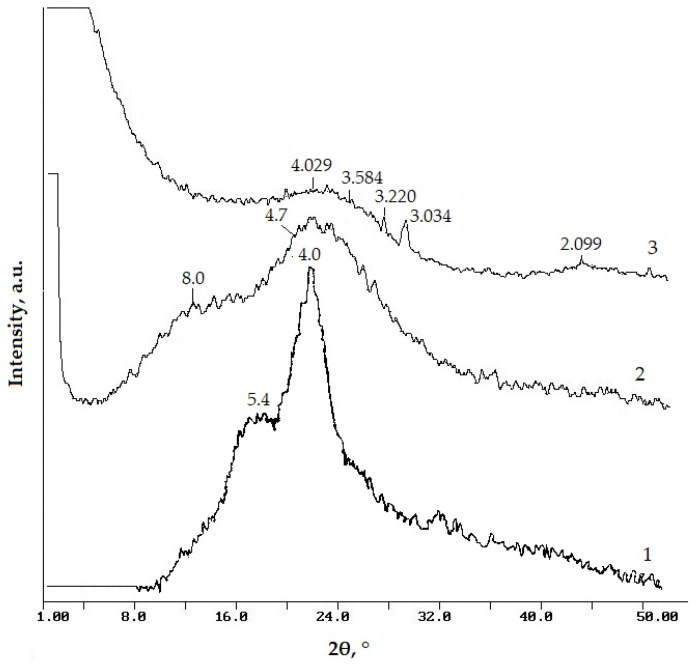
XRD patterns of RH (1), SCC (2), and SCC-V (3).

Rice husk was obtained from the village of Bakanas, Almaty Region. The total chemical and elemental compositions of rice husk are shown in Table 1 and Table 2. From the presented data, it follows that a large part of the rice husk (more than 40 wt.%) is formed by carbon. Silicon dioxide accounts for 20 wt.% The remaining elements are present in insignificant amounts. The presence of only crystalline cellulose was recorded using the method of X-ray diffraction analysis of the crystalline phases in the rice husk (Figure 1). Crystalline cellulose is identified according to reflections at angles 2θ = 22° (reflection 002) with d_1_~4.0 Å and 16° (reflections 101 and 101 are not resolved) with d_2_~5.4 Å [31]. Reflections of any crystalline modifications of silicon dioxide are absent. This indicates that silicon dioxide in rice husk is contained in an amorphous form. The rice husk was ground to a fraction of <1 mm for use in the high-temperature material production as a source of silicon dioxide and a burnout additive.

**Figure 2:**
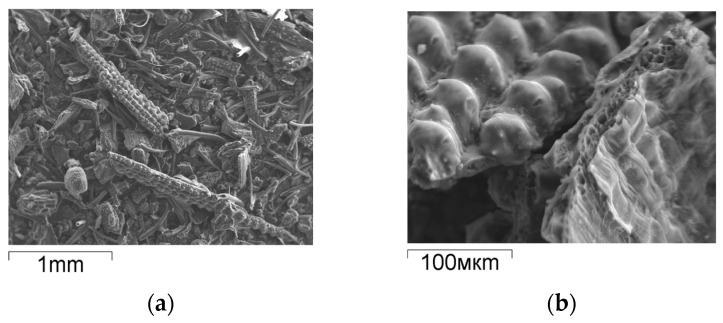
SEM images: (**a**) RH; (**b**) SCC.

As can be seen from the SEM image (Figure 2a), rice husk has a fibrous structure. The fibers on both sides are covered with surface layers. The inner surface layer looks like a protective film. The outer surface layer resembles the structure of a corncob. The surface topology of the rice husk is preserved after pyrolysis (Figure 2b). At the fracture site of the pyrolyzed particle (Figure 2b), voids are visible between the carbon fibers, forming the porous structure of the material.

**Figure 3:**
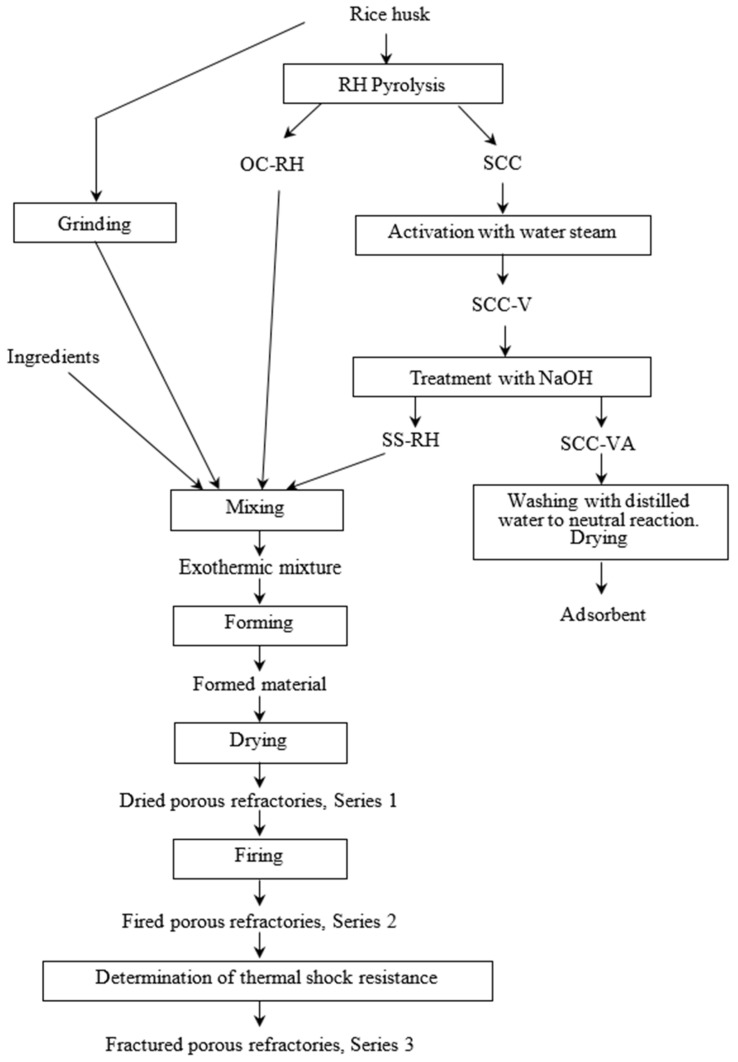
Technological scheme of complex processing of rice husk.

The organic condensate and sodium silicate solution were obtained from the production of thermal processing of rice husk (Figure 3). Using the method of thermal analysis, we previously established [30] that the thermal destruction of rice husk proceeds at a maximum rate at 235–265 °C. At a temperature of 300 °C, the formation of new substances with heat release is observed. At temperatures above 450 °C, endothermic and exothermic processes begin, associated with the formation of a carbon structure. A temperature of 450 °C and a duration of 30 min are favorable conditions for the pyrolysis of rice husk without air access in order to obtain a silica–carbon composite as a raw coal for the production of a carbon sorbent. Firstly, as previously shown using the Electron Paramagnetic Resonance method, the silica–carbon composite has the largest number of paramagnetic centers (1.0 × 10^19^ spin g^−1^) at this temperature. In this state, SCC is most active due to the presence of a large number of free radicals. It is at this temperature that the complete thermal destruction of rice husk biomass ends. Then, the pyrolysis product (SCC) is activated with water steam at 850 °C. As established in previous studies [32], the silica–carbon composite after pyrolysis at 400 °C has a specific surface area of ~70 m^2^ g^−1^. The specific surface area of the silica–carbon composite obtained at 600 °C is slightly higher. It reaches a value of 150 m^2^ g^−1^. However, after activation with water steam, both materials are characterized by equal values (300–310 m^2^ g^−1^) of specific surface area.

The chemical and elemental compositions of SCC are presented in Table 1 and Table 2. From the given data, it follows that as a result of pyrolysis, the SiO_2_ content in the silica–carbon composite practically doubles compared to the initial rice husk.

Two amorphous halos are recorded at angles 2θ ~12° and ~20° on the X-ray diffraction pattern of rice husk pyrolyzed at 450 °C (SCC, Figure 1), characteristic of amorphous carbon materials. The halo with d~8.0 Å indicates the presence in the analyzed material of a hydrocarbon phase with an unestablished structure. The halo with d~4.7 Å indicates the presence of a polynaphthenic phase [31]. Two halos are recorded at angles 2θ ~24° and ~45° on the X-ray diffraction pattern of the silicon–carbon composite activated with water steam at 850 °C (SCC-V, Figure 1). It is known that the halo with d_002_~3.584 Å at an angle 2θ ~24° corresponds to the presence of a graphite-like phase. The appearance of a halo in the region 2θ ~45° indicates the formation of a two-dimensionally ordered (turbostratic) carbon structure [31]. According to X-ray diffraction analysis data (Figure 1), silicon dioxide also undergoes changes after activation of SCC with water steam. The cristobalite phase (d_002_~4.0 Å) is identified on the X-ray diffraction pattern of the SCC-V sample.

During the pyrolysis of rice husk, an organic condensate is formed upon condensation of the outgoing vapors (OC-RH, Figure 3). According to GC-MS data (Figure S1 and Table S1), OC-RH is an aqueous solution of a mixture of organic compounds (pH 4.5). The group composition (by classes of organic compounds) of the organic condensate is as follows: carboxylic acids—23%, phenols—13%, ketones—12%, cyclic aliphatic hydrocarbons—6%, heterocyclic compounds—4%, alcohols and ethers—2%, unidentified substances—15%, water content—25%. In the present study, OC-RH was used as a binder instead of lignosulfonate, a traditional ingredient for obtaining high-temperature materials.

A silicate solution (SS-RH) was used as a second binder. Its formation is shown in the Scheme (Figure 3) in the section outlining the production of a carbon sorbent based on a silica–carbon composite from rice husk. As can be seen from the scheme in Figure 3, SCC was activated with water steam at 850 °C for 30 min to obtain the SCC-V product. SCC-V was treated with a 70 g dm^−3^ sodium hydroxide solution at a solid (g)/liquid (cm^3^) (S/L) ratio of 1:10 with boiling for 90 min. The resulting SS-RH solution was used in the present study to obtain experimental high-temperature materials instead of commercial liquid glass. The sodium silicate content in SS-RH, calculated on a dry basis, was ~6 wt.%

The following ingredients for preparing the high-temperature material mixtures were purchased from wholesale and retail suppliers in accordance with technical documentation:

Refractory chamotte clay (RCC), TU 1522-009-00190495-99. The chemical composition of the clay is given in Table 3. The fraction was 0.06–1 mm.

- Content, wt.%: Al_2_O_3_—at least 30. The fraction was 0.06–2.0 mm.
- Sodium liquid glass, GOST 13078-2021 [33]. Content, wt.%: SiO_2_—24.8–36.7; Na_2_O—8.1–13.3.
- Technical liquid lignosulfonate, TU 2455-028-00279580-2014. Content, wt.%: dry substance—50; sodium salt of lignosulfonic acid—32; pH—4.6.
- Magnesium sulfate 7-hydrate, GOST 4523-77 [34]. Crystalline powder. Content, wt.%: MgSO_4_·7H_2_O—99.
- PAP-1 grade aluminum powder, GOST 5494-2022 [35]. Impurity content, wt.%: Fe ≤ 0.5; Si ≤ 0.4; Cu ≤ 0.05; Mn ≤ 0.05. The fraction was <8 μm.

### 2.2. Analytical Methods

The chemical composition of the RH and SCC samples was performed by analyzing the ash fraction after removing volatile matter at 1000 °C. Ash decomposition was carried out with aqua regia. In the resulting solutions, Ca, Mg, Mn, Pb, Cu, and Zn were determined using atomic absorption spectrometry (Agilent AA240FS, Agilent Technologies, Santa Clara, CA, USA); SiO_2_ was determined with gravimetric analysis; Al_2_O_3_ and Fe were determined using volumetric analysis; and TiO_2_ and P were determined via colorimetric analysis (KFK-3-01, JSC “Zagorsk Optical and Mechanical Plant”, Sergiev Posad, Russia). K and Na were determined using flame photometry (FPA-2-01, Sergiev Posad, Russia).

The composition of the RCC sample was determined using XRF analysis with a portable X-ray fluorescence spectrometer Niton XL3t (Thermo Scientific Portable Analytical Instruments Inc., Tewksbury, MA, USA).

Elemental analysis was performed with a CHNOS Vario MICRO Cube elemental analyzer (Elementar Analysensysteme GmbH, Hanau, Germany).

X-ray diffraction patterns of the RH, SCC, and SCC-V samples were determined using a DRON-2 computerized diffractometer with modernized collimation using filtered CuKα radiation in reflected rays in a 17 × 17 × 1.5 mm metal cell [31]. X-ray diffraction analysis of high-temperature materials was conducted on an automated DRON-3 diffractometer with CuKα radiation and a β-filter. X-ray phase analysis on a semi-quantitative basis was performed with diffractograms of powder samples using the method of equal weights and artificial mixtures. Quantitative ratios of crystalline phases were determined. Diffractogram interpretation was carried out using ICDD card file data: PDF 2 (Powder Diffraction File) Release 2022 database and HighScorePlus software vers. 3.0e (3.0.5)/30 January 2012.

Scanning electron microscopy of RH and SCC was performed on the Superprobe 783 microanalyzer (JEOL Ltd., Tokyo, Japan). Photography of secondary electrons was performed by using an INCA Energy Dispersive Spectrometer (Oxford Instruments, London, UK). To avoid the formation of a charge on the analyzed materials, which are capable of deflecting the electron beam, the samples were precoated with a thin, structureless gold film in a fine-coat ion-sputtering apparatus (JEOL, Tokyo, Japan). SEM and microanalysis of refractories were performed on the JEOL JXA electron-probe microanalyzer 8230 (JEOL Ltd., Tokyo, Japan) at an accelerating voltage of 20 kV, with electron beam current up to 20 nA. SEM studies were conducted in backscattered electron mode (COMPO). Microanalysis was performed in energy-dispersive spectroscopy (EDS) mode. Additionally, EDS elemental mapping was conducted in accordance with references [36,37,38].

The particle distribution by fractions (sizes) was determined using the sieve analysis method.

Thermal analysis (DTA/TG) was carried out on a 449 F3 Jupiter synchronous thermal analyzer (STA; NETZSCH, Selb, Germany). Before heating, the furnace chamber was evacuated (up to ~92% of the total volume) and then purged with inert gas (Ar) for 5 min. Heating was performed at a rate of 15 °C min^−1^ in a high-purity argon atmosphere. The total gas flow rate was maintained within 120–130 cm^3^ min^−1^. The results obtained using the STA 449 F3 Jupiter were processed using NETZSCH Proteus software for Thermal Analysis, vers. 5.1.0/24 November 2009.

The chemical composition of OC-RH was determined using a gas chromatograph with a GC/MS Agilent 6890 N/5973 N mass-spectrometric detector (Wilmington, DE, USA). The sample volume was 0.2 μL with a split ratio of 50:1, and the injector temperature was 250 °C. The separation was performed on a DB-XLB column 30 m long with an internal diameter of 0.25 mm and a film thickness of 0.50 μm in constant flow mode at a carrier gas (helium) speed of 1 cm^3^ min^−1^. The column oven temperature program was set as follows: 40 °C, hold 10 min; ramp at 10 °C min^−1^ to 150 °C, hold 4 min; and ramp at 20 °C min^−1^ to 250 °C, hold 30 min. The mass-spectrometric detection was performed in total ion current mode in the m/z range 10–300. The interface temperature was 280 °C.

### 2.3. Sample Preparation

To produce high-temperature materials, a known mixture composition, developed by scientists at the RSE Astana Branch of the National Center on Complex Processing of Mineral Raw Materials of the Republic of Kazakhstan, was used as a control (AlMgC). This mixture is used for the production of refractories used in metallurgical enterprises. AlMgC contains the following (wt.%, Table 4): MgSO_4_·7H_2_O—4.5; Al powder—2; refractory clay—59; liquid glass—8.5; lignosulfonate—2; chamotte—24.

Based on this composition, experimental samples were prepared using RH, SS-RH, and OC-RH in various mixes (Table 4). The composition of the AlMgC-SS8.5 sample was analogous to the AlMgC sample. However, in the experimental AlMgC-SS8.5 sample, the commercial liquid glass was replaced with an equal amount (8.5 wt.%) of SS-RH solution. To prepare the AlMgC-RH2.4 sample, in the control composition (AlMgC), the liquid glass was replaced with an equal (8.5 wt.%) amount of SS-RH solution, the lignosulfonate (2 wt.%) was replaced with the same amount of OC-RH solution, the chamotte content was reduced, and 2.4 wt.% of rice husk was introduced instead. In AlMgC-RH14.4, instead of liquid glass and lignosulfonate, 10.5 wt.% of OC-RH was used, and instead of 24 wt.% of chamotte, 14.4 wt.% of rice husk was introduced. This content of RH was determined based on the analysis of the literature data [7]. Taking into account the low bulk density of rice husk (0.1 g·cm^−3^) and its large volumetric content in the mixture, the amounts of clay (up to 67.1 wt.%), magnesium sulfate heptahydrate (up to 5 wt.%), and aluminum powder (up to 3 wt.%) were increased (Table 4).

Samples C and C-RH14.4 were prepared without the use of magnesium sulfate heptahydrate and aluminum powder (Table 4). They were used for comparative thermal analysis.

The procedure for producing high-temperature materials was as follows (Figure 3). To prepare the control sample AlMgC, all ingredients were weighed on scales in the amounts indicated in Table 4. First, magnesium sulfate heptahydrate, aluminum powder, refractory clay, and chamotte were mixed. Then, lignosulfonate and liquid glass were introduced into the mixture of dry ingredients. The mixture was thoroughly mixed and sent for forming. The preparation of the experimental sample mixtures was carried out analogously to the control sample. Commercial ingredients (liquid glass and lignosulfonate) were replaced with rice husk processing products (SS-RH and OC-RH), as shown in Table 4. Ground rice husk was introduced instead of chamotte into the AlMgC-RH2.4 and AlMgC-RH14.4 samples after the preliminary mixing of dry and liquid ingredients.

**Figure 4:**
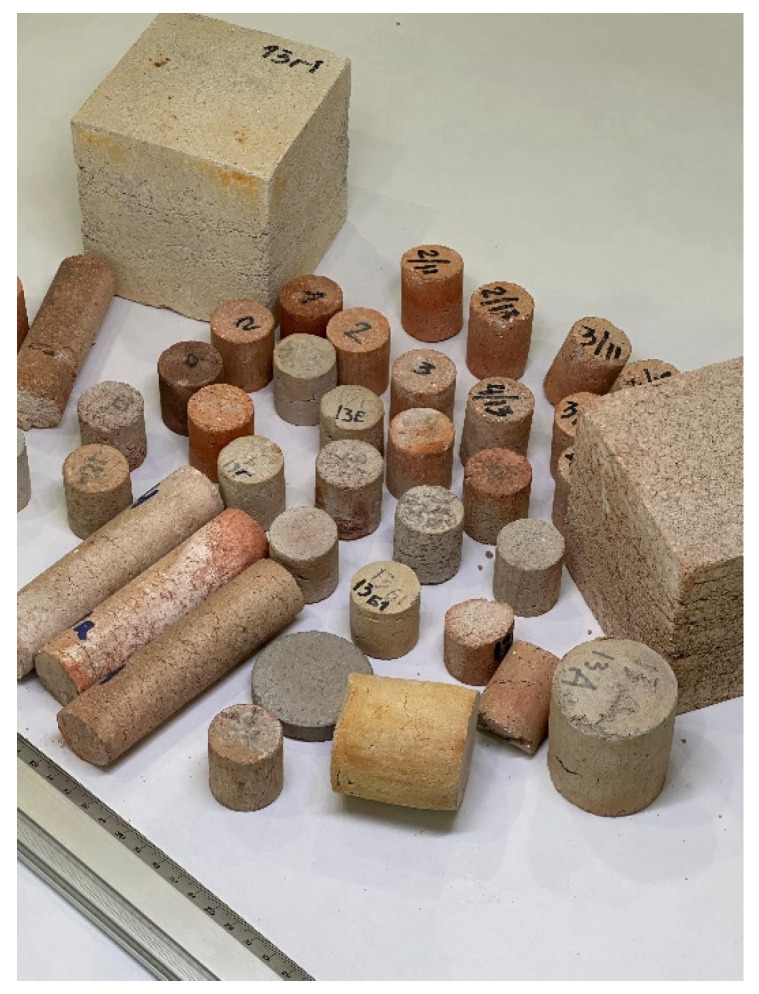
High-temperature materials of various shapes before and after testing.

High-temperature material samples were formed in molds of sizes 30 × 30 mm, 50 × 60 mm, 50 × 10 mm, 30 × 100 mm, and 100 × 100 × 100 mm (depending on the requirements for measuring their mechanical and thermal properties, Figure 4). The samples were air-dried for 24 h then dried in a drying oven at 150 °C for 24 h. The dried samples were labeled as Series 1 (Figure 3). The dried samples were heated to 950 °C and held at this temperature for 1 h in an air atmosphere. After sintering, the samples were kept in the air until completely cooled and then tested. The fired samples were labeled as Series 2 (Figure 3).

In the present study, the refractory material samples were labeled in accordance with the compositions of the mixtures, as indicated in Table 4, and their level of readiness:

- Dried, Series 1: 1-AlMgC, 1-AlMgC-SS8.5, 1-AlMgC-RH2.4, and 1-AlMgC-RH14.4.
- Fired, Series 2: 2-AlMgC, 2-AlMgC-SS8.5, 2-AlMgC-RH2.4, and 2-AlMgC-RH14.4.
- Destroyed during the thermal shock resistance test (description is given in the Section 2.4), Series 3: 3-AlMgC, 3-AlMgC-SS8.5, 3-AlMgC-RH2.4, and 3-AlMgC-RH14.4.

### 2.4. Testing and Analysis

The apparent porosity, volumetric weight, and true density of the Series 2 samples, size 30 × 30 mm, were determined in accordance with the standardized procedures specified in the interstate standard GOST 2409-2014 “Refractories. Method for determination of bulk density, apparent and true porosity, and water absorption [39]”.

The volumetric weight (*W_V_*, g·cm^−3^) of the refractories was determined as the ratio of the initial mass of the dry sample to its volume:

(1) $$W_{v}=\frac{m_{1}}{V}$$

where *m*_1_ is the mass of the dry sample (g), and *V* is the volume of the sample (cm^3^).

The apparent porosity (*P_a_*, g·cm^−3^) of the refractories was determined as the ratio of the increase in the mass of the sample after its three-hour boiling in water to its volume:

(2) $$P_{a}=\frac{m_{2}-m_{1}}{V}\cdot 100\%$$

where *m*_1_ is the mass of the dry sample before boiling (g), *m*_2_ is the mass of the wet sample after boiling (g), and *V* is the volume of the sample (cm^3^).

The true density (*ρ*, g·cm^−3^) of the refractories was calculated from the mass of the analytical sample and its true volume, which was determined using a pycnometer with water:

(3) $$\rho =(\frac{m_{1}}{m_{1}-m_{2}}+\alpha )\cdot \rho_{H_{2}O,}$$

where *m*_1_ is the mass of the dry sample (g), *m*_2_ is the mass of the impregnated sample immersed in water (g), *α* is a correction factor that takes into account the degree of pore saturation and water temperature, and *ρ* is the density of distilled water at the thermostatic temperature (g·cm^−3^).

The compressive strength of the Series 2 samples was determined in accordance with the interstate standard GOST 4071.1-2021 “Refractory products with less than 45% true porosity. Method for determination of compressive strength at room temperature [40]”. The test sample with a size of 100 × 100 × 100 mm at room temperature was loaded uniformly and continuously at a rate of (2 ± 0.2) N (mm^2^ s)^−1^ until failure (Figure S2a–c). The ultimate compressive strength (*σ*, MPa) at room temperature was calculated from the maximum load measured at the failure of the sample and the average area of its cross-section, to which the load was applied:

(4) $$\sigma =\frac{F_{\max}}{s}$$

where *F_max_* is the maximum load measured at the failure of the sample (N), *s* is the average area of its cross-section (mm^2^). The determination of the interfacial bond strength (adhesion) of the Series 2 samples with a substrate, or pull-off strength, was performed according to the interstate standard GOST 31356-2007 “Dry building mixtures on a cement binder. Test methods [41]”. The interfacial bond strength (adhesion, Ai, MPa) was determined using the pull-off force of a 50 × 10 mm sample glued with epoxy adhesive to a concrete slab (Figure S2d). The tests were conducted at room temperature. The loading rate was (250 ± 50) N s^−1^. The bond strength (*A_i_*, MPa) was calculated using the following formula:

(5) $$A_{i}=\frac{F}{S},$$

where *F* is the maximum pull-off force of the sample from the base (N), and *S* is the contact area of the sample surface with the base (mm^2^).

Testing of Series 2 samples to determine the tensile strength in bending was carried out according to GOST 10180-2012 “Concretes. Methods for strength determination using reference specimens [42]”. The tensile strength in bending (*R_tb_*, MPa) was determined in a testing machine by loading cylindrical samples of size 30 × 100 mm uniformly and continuously until failure at a constant loading rate of (0.05 ± 0.01) MPa s^−1^ (Figure S2e) and was calculated using the following formula:

(6) $$R_{tb}=\delta \frac{Fl}{ab^{2}}K_{W},$$

where *F* is the breaking load (N); *a*, *b*, and *l* are the width, height of the sample, and the distance between the supports (mm), respectively; *δ* is a scale factor for converting the strength of the sample to the strength of standard size and shape samples; and *K_W_* is a correction factor that takes into account the moisture content of the samples at the time of testing.

The thermal shock resistance (number of thermal cycles) of the Series 2 samples was determined according to the ability of the samples to withstand cyclic temperature changes (heating–cooling) in at least three repetitions. For this, the 30 × 30 mm sample was heated to 850 °C for 40 min and then cooled in a vessel with water at 15 °C for 3 min and in the air at room temperature for 7 min until the sample lost >20% of its initial mass. The destroyed samples were labeled as Series 3 (Figure 3).

The thermal conductivity was determined according to the interstate standard GOST 30256-94 “Building materials and products. Method for determination of thermal conductivity by cylindrical probe [43]”. The determinations were carried out on 100 × 100 × 100 mm samples with a cylindrical probe (Figure S2g). The thermal conductivity (*λ*, W (m·K)^−1^) was calculated using the following formula:

(7) $$\lambda =0.05516\;I^{2}R\;E_{0}/\Delta E,$$

where *I* is the probe heating current (A), *R* is the resistance of the probe heater (Ohm m^−1^), *E*_0_ is the sensitivity of the probe thermocouple (mkV K^−1^), and Δ*E* is the increase in the EMF of the probe thermocouple (mkV). Δ*E* was calculated as the difference between the arithmetic mean values of the EMF measured in the time intervals of 8–12 and 4–6 min.

The deformation of samples under load with the increase in temperature was determined according to the interstate standard GOST 4070-2000 “Refractory products. Method for determination of refractoriness under load [44]”. The measurements were performed on cylindrical Series 2 samples, size 50 × 60 mm, with a hole of 13 mm diameter drilled along the axis in the direction of brick pressing. During the experiments, the relative change in the height of the high-temperature material samples under the combined effect of a static mechanical load (0.05 N mm^−2^), increasing temperature (up to 1100 °C), and time (heated to 700 °C at a rate of 10 °C min^−1^ and from 700 to 1100 °C heated at a rate of 3 °C min^−1^) was determined. During the test, the temperature (T_max_) corresponding to the maximum expansion of the sample, the softening start temperature (T_0_._6_) corresponding to a 0.6% reduction in the sample height from the maximum expansion, and the temperature (T_4_) corresponding to a 4% reduction in the sample height from the maximum expansion were recorded.

## 3. Results and Discussion

### 3.1. X-Ray Diffraction Analysis

**Figure 5:**
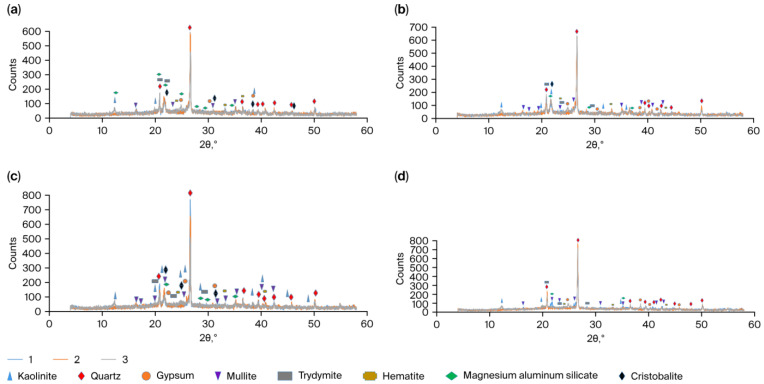
XRD patterns of the Series 1–3 high-temperature material samples: (**a**) AlMgC; (**b**) AlMgC-SS8.5; (**c**) AlMgC-RH2.4; (**d**) AlMgC-RH14.4.

The samples of high-temperature materials prepared in accordance with the compositions presented in Table 1, after drying (Series 1), sintering (Series 2), and destruction during the thermal shock resistance test (Series 3), were studied using X-ray diffraction analysis. According to the data obtained (Figure 5a–d), all samples from Series 1 (1-AlMgC, 1-AlMgC-SS8.5, 1-AlMgC-RH2.4, и 1-AlMgC-RH14.4) contain quartz, tridymite, hematite, mullite, and magnesium aluminosilicate.

After sintering, in samples 2-AlMgC, 2-AlMgC-SS8.5, and 2-AlMgC-RH2.4, the crystallization of tridymite is observed with the formation of a cristobalite phase (Figure 5a–c). This does not occur in sample 2-AlMgC-RH14.4, i.e., after the replacement of chamotte with rice husk. In RH, silicon dioxide is initially present in an amorphous form (Figure 1). In this sample, only quartz reflections were detected among the crystalline phases. The formation of cristobalite upon replacing clay with silica from rice husk was observed by the authors of [22] during the production of refractory ceramics at 1300 °C. They also showed that silica from rice husk does not participate in the reaction of mullite formation. In the present study, the high-temperature materials were sintered at 950 °C. The phase transition of amorphous silica to cristobalite and the formation of mullite were not observed.

Complete preservation of all crystalline phases was observed in Series 3 samples upon thermal degradation of the high-temperature materials after iterative heating–cooling in water cycles: quartz, cristobalite, mullite, hematite, and magnesium aluminosilicate phases were detected in samples 3-AlMgC, 3-AlMgC-SS8.5, 3-AlMgC-RH2.4, while sample 3-AlMgC-RH14.4 demonstrated the presence of the quartz phase only. Reflections of gypsum (CaSO_4_·xH_2_O) and kaolinite (Al_2_(Si_2_O_5_)(OH)_4_) appear in the diffractograms of all these samples (Figure 5a–d). This indicates the occurrence of a degradation process of some phases in the studied materials. However, it proceeds independently of the replacement of traditionally used ingredients (chamotte, liquid glass, and lignosulfonate) with new components (rice husk, sodium silicate solution from rice husk, and organic condensate from rice husk pyrolysis). This is consistent with the conclusion of [7], which states that plant-based additives do not affect the phase composition during the production of thermal insulating materials using clay.

### 3.2. Thermal Analysis

**Figure 6:**
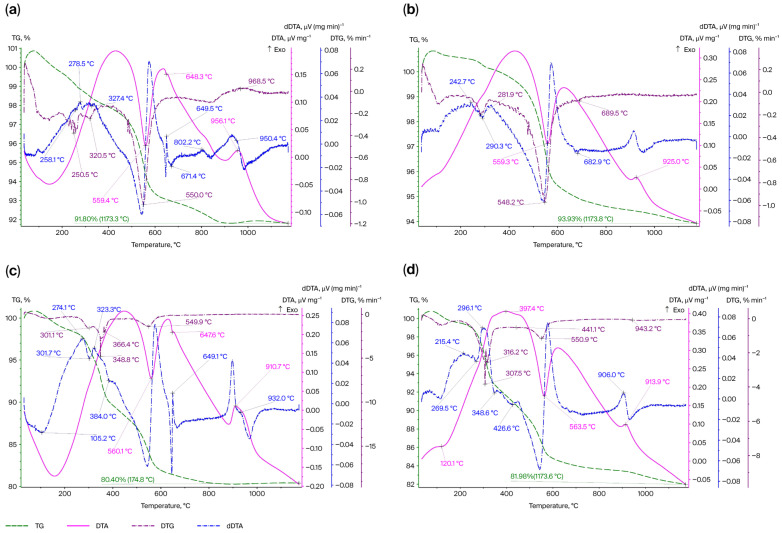
Thermograms of high-temperature materials: (**a**) 1-AlMgC; (**b**) 1-C; (**c**) 1-AlMgC-RH14.4; (**d**) 1-C-RH14.4.

To interpret the thermal effects, a thermal analysis of the new (experimental) mixture (1-1-AlMgC-RH14.4) was performed and included a comparison with the control mixture (without the addition of rice husk and organic condensate from rice husk pyrolysis, 1-AlMgC). Samples obtained from the experimental and control mixtures but not containing Al and MgSO_4_·7H_2_O (1-C-RH14.4 and 1-C, respectively) were subjected to TA. On the DTA curve of samples 1-AlMgC and 1-AlMgC-RH14.4, in the 100–200 °C range, a broad endothermic effect is observed, accompanied by a slight (1–2%) mass loss (Figure 6a,c). Since samples C-RH14.4 and 1-C lose hygroscopic moisture at 100–120 °C (Figure 6b,d), the endothermic effect on the DTA curve of samples 1-AlMgC and 1-AlMgC-RH14.4 with an extremum at 150 °C is also explained by the dehydration of magnesium sulfate. A series of thermal effects of different directions is observed on the dDTA curve of all samples in the temperature range of 100–400 °C. This may reflect the simultaneous occurrence of several processes, including the dehydration of mineral components of the clay and the decomposition of organic compounds (RH and OC-RH).

The exothermic effect of sample 1-C-RH14.4 at ~397 °C is caused by the presence of the organic component. It is known that during the carbonization of rice husk in this temperature range [30], new substances are formed, and condensation processes accompanied by mass loss on the TG curve occur in the resulting carbon residue. Judging by the characteristics of the DTG curve of sample 1-C-RH14.4, its mass loss proceeds at a maximum rate at 307 °C. This process is less pronounced in sample 1-AlMgC-RH14.4, which contains Al and MgSO_4_·7H_2_O.

A sharp endothermic effect with an extremum at ~560 °C and a rather intense exothermic effect in the range of (depending on the composition of the sample) 910–950 °C are recorded on the DTA curves of all four samples (Figure 6). The combination of these effects can be interpreted as a manifestation of the aluminosilicate. In the region of the effect at ~560 °C, the release of water associated with hydroxyl groups and the amorphization of the substance occur. The peak at 910–950 °C reflects the crystallization of amorphous phases. Judging by the characteristics of the dDTA curves, this process is more intensive in sample 1-AlMgC-RH14.4. This sample, unlike all others, exhibits a sharp, intense peak in the 900 °C region, which is not accompanied by a change in mass. In general, in sample 1-AlMgC-RH14.4, the mass change does not occur after 800 °C.

Among the series of weaker thermal effects recorded on the dDTA curves, two exothermic effects that appeared only in samples 1-AlMgC-RH14.4 (at 649 and 932 °C) and 1-AlMgC (at 649 and 802 °C) and endothermic effects with extrema at 671 °C (1-AlMgC), 667 °C (1-AlMgC-RH14.4), 682 °C (1-C), and 677 °C (1-C-RH14.4) are noteworthy. The latter may reflect the decomposition of the magnesium chlorite lattice Mg_4_(Mg,Al)_2_[AlSi_3_O_10_](OH)_8_ with the release of structural water. It is noteworthy that their intensity is higher in samples 1-AlMgC and 1-AlMgC-RH14.4, i.e., those initially containing Al and MgSO_4_·7H_2_O. The exothermic effect at 802 °C in sample 1-AlMgC is due to the formation of a mineral with an olivine structure (forsterite) as a result of the interaction of active MgO and SiO_2_ oxides [45]. As indicated in [45], spinel is formed upon its further heating. The formation of spinel (MgAl_2_O_4_) was observed by the authors of [28,46] during the production of refractories using silica from rice husk with firing of the initial mixtures at 1050–1170 °C. Subsequent elevation of the sintering temperature (≥1230 °C) leads to cordierite phase formation [28]. While spinel presence was registered at 1230 °C in reference [27], it was observed under conditions employing alumina additives in the range of 5 to 30 wt.%. The findings indicate that progressive increases in alumina content enhance spinel formation while inhibiting cordierite crystal development. The exothermic effect at 932 °C in sample 1-AlMgC-RH14.4, not accompanied by a change in mass on the TG curve, as noted above, may reflect the interaction between the components of the sample. Evidently, it should be considered in conjunction with the weak endothermic effect with an extremum at 647 °C, which appeared on the descending branch of the main endothermic effect on the DTA curve. It is very close to the melting temperature of aluminum (660 °C) and may reflect the softening process of MgSO_4_ in the presence of Al. The local chemical interaction at the interface of these components requires energy absorption before the main exothermic reaction is initiated [4]:

(8) $${MgSO}_{4}+2Al\to MgO\cdot {Al}_{2}O_{3}+S,$$

(9) $$3{MgSO}_{4}+2Al\to 3MgO+{Al}_{2}O_{3}+3{SO}_{2}↑.$$

The authors of [5], comparing the aluminum–chamotte and aluminum–chamotte–magnesium sulfate systems, observed exothermic effects during the combustion of the charge with magnesium sulfate in the 180–660 °C range and at 800 °C. Accordingly, the exothermic effect with a peak at 649 °C may correspond to the beginning of exothermic reactions that develop upon further heating. Due to the presence of organic components, this process in the experimental mixture 1-AlMgC-RH14.4, as shown above, proceeds at 932 °C, unlike in sample 1-AlMgC (802 °C). This is because the burnout of organic components is associated with the formation and removal of gaseous compounds, which inevitably leads to the formation of a porous structure [7]. According to the TG curves (Figure 6a,c), the residual mass of sample 1-AlMgC-RH14.4 at 1174 °C was 80.4%, which is almost 11.5% lower than that of sample 1-AlMgC at the same temperature. The increase in porosity causes a shift in the exothermic reaction upon heating the mixture to a higher temperature zone due to the slowing of heat transfer. This conclusion is supported by the observations of the authors of [5] that the formation of gaseous substances suppresses the combustion rate of the aluminum–chamotte–magnesium sulfate mixture. Nevertheless, by taking the mass loss indicator as the completion of the combustion reaction, it can be said that this process ends within 900 °C in the case of samples 1-AlMgC and 1-AlMgC-RH14.4, unlike in samples 1-C and 1-C-RH14.4, where the mass reduction in the samples proceeds throughout the entire studied temperature range (i.e., up to 1174 °C) as seen from the corresponding TG curves.

**Figure 7:**
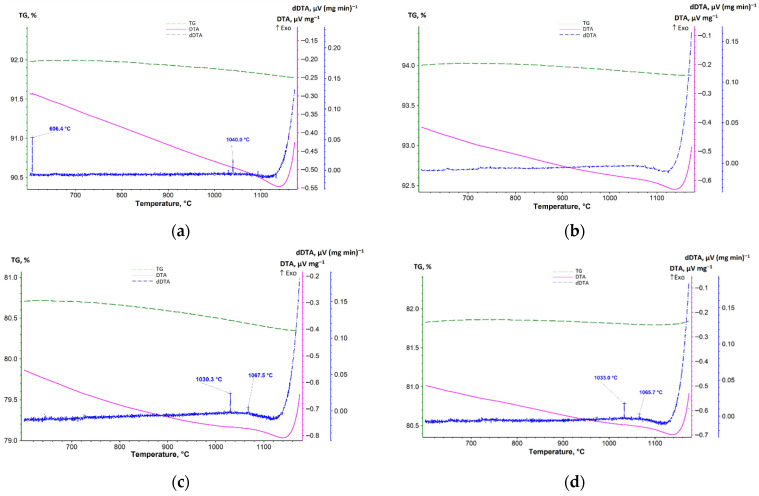
Cooling curves of high-temperature materials: (**a**) 1-AlMgC; (**b**) 1-C; (**c**) 1-AlMgC-RH14.4; (**d**) 1-C-RH14.4.

Two low-intensity exothermic effects were observed on the dDTA curves of samples 1-AlMgC, 1-AlMgC-RH14.4, and 1-C-RH14.4 upon cooling at a rate of 17 °C/min (Figure 7a,c,d). On the dDTA curve corresponding to the control sample 1-AlMgC, the peaks of these effects are at 1040 °C (less intense) and 606 °C (more intense). These effects, which reflect phase transformations with heat release, are most likely caused by the recrystallization of oxide or silicate phases. On the dDTA curves of samples containing a plant-based additive, exothermic effects were recorded at 1067 (less intense) and 1030 °C (more intense) for sample 1-AlMgC-RH14.4 and at 1065 (less intense) and 1033 °C (more intense) for sample 1-C-RH14.4. The presence of these exothermic effects in samples with a plant-based additive, regardless of the introduction of aluminum and magnesium sulfate into the initial composition, provides a basis for assuming that they are caused by the graphitization process of the carbon residue [31]. Theoretically, this process is possible as the thermal analysis was performed in an inert atmosphere. The metal oxides present in the clay, as shown in references [47,48], are catalysts for the formation of the carbon structure. No thermal effects were recorded on the dDTA curve of sample 1-C (Figure 7b).

### 3.3. Scanning Electron Microscopy

**Figure 8:**
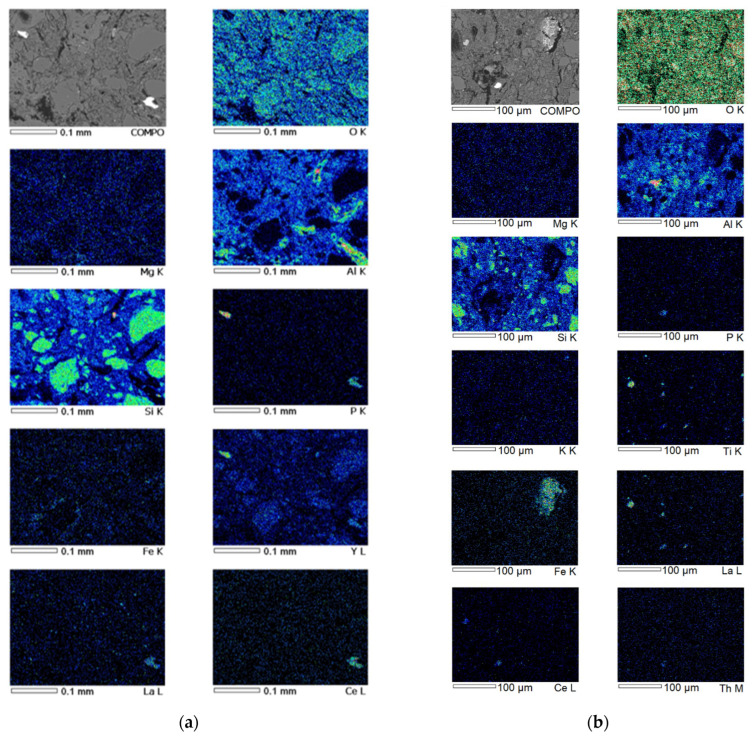
SEM images and EDS elemental mapping of the high-temperature materials: (**a**) 2-AlMgC; (**b**) 2-AlMgC-RH14.4.

During the SEM study of samples 2-AlMgC and 2-AlMgC-RH14.4, oxide particles were identified as the main micro-objects on the surfaces of both polished sections (Figure 8). Primarily, particles of quartz and aluminosilicates were observed. Particles of a heavy fraction were registered on the surface of the polished section of sample 2-AlMgC-RH14.4, such as titanium oxides (including those with iron content), zircon, iron with common impurities (magnesium, aluminum, and silicon), as well as lanthanum and cerium phosphate with thorium impurity (Figure 8b, Figures S3 and S4).

The presence of particles of oxide compounds of zirconium, titanium (with iron content), and iron was also established (Figure 8a) on the surface of the polished section of sample 2-AlMgC. An oxygen-free particle was also found here. It is an agglomerate of three compounds of the Al-Si-Fe and Al-Si-Ti-V types and nominally “pure” silicon. Particles of rare earth elements were identified via mapping and subsequent point microanalysis (Figures S5 and S6): lanthanum and cerium phosphate with a thorium impurity, as in the previous sample, and gadolinium, ytterbium, and dysprosium phosphate with an yttrium impurity. Within the framework of this study, the assessment of the presence of metal oxides in the studied mixtures is important as evidence of the existence of potential catalysts for the processes discussed above.

**Figure 9:**
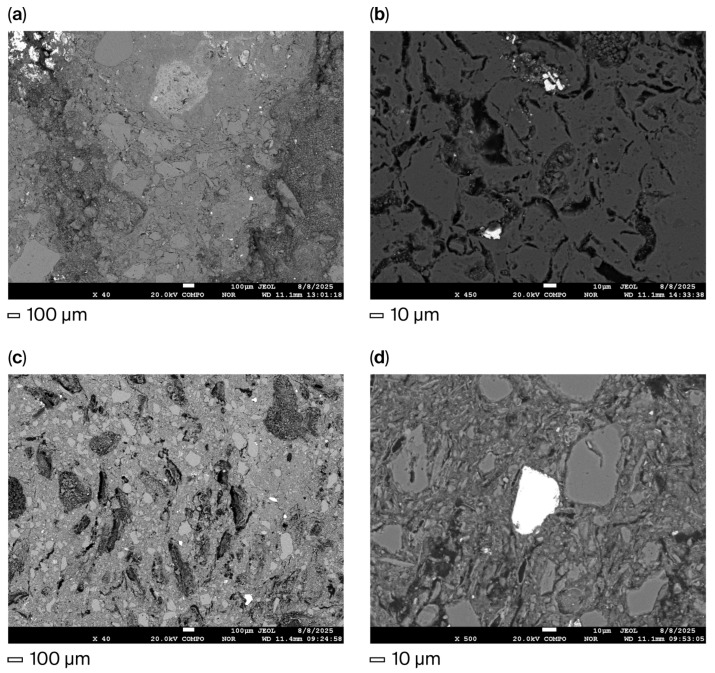
Microstructure of high-temperature materials: (**a**) 2-AlMgC×40; (**b**) 2-AlMgC×450; (**c**) 2-AlMgC-RH14.4×40; (**d**) 2-AlMgC-RH14.4×500.

The microstructural state is of particular interest as it largely determines the performance characteristics of high-temperature materials. The surfaces of the polished sections of the control (2-AlMgC) and experimental (2-AlMgC-RH14.4) samples, as seen in Figure 9, are characterized by a large number of cavities remaining after the dislodging of unidentified particles or fragments of high-temperature materials. Despite the fact that both samples are composed of particles of different sizes, ranging from less than 1 µm to 100 µm and larger, sample 2-AlMgC is distinguished by denser packing. Sample 2-AlMgC-RH14.4 appears looser. It is characterized by the presence of narrow and long rod-shaped particles, which are distributed along the surface of the polished section. Both samples have a developed pore system. However, unlike sample 2-AlMgC, the pores of sample 2-AlMgC-RH14.4 contain an embedded porous framework, formed, it seems, by microfibers after the destruction of the rice husk in places where volatile substances were removed.

### 3.4. Performance Properties

#### 3.4.1. Physical and Mechanical Properties

As shown in Table 5, the contents of organic components directly affect the porosity, density, and volumetric weight of the resulting high-temperature materials. In the control sample 2-AlMgC, the formation of the porous structure results from the use of magnesium sulfate heptahydrate, liquid lignosulfonate, and sodium silicate (Table 4). In the preparation of sample 2-AlMgC-SS8.5, which is nearly analogous to sample 2-AlMgC, the same amount of magnesium sulfate heptahydrate (4.5%) was used. However, instead of 2% lignosulfonate, 2% organic condensate obtained during rice husk pyrolysis was added to the mixture, and the commercial liquid glass was replaced with an equal amount (8.5%) of sodium silicate solution synthesized from rice husk. Both samples are characterized by the same value of apparent porosity (34%). The replacement of chamotte in sample 2-AlMgC-RH2.4 with an equal amount of rice husk (2.4%) contributed to an increase in apparent porosity to 36%. The maximum value of this indicator (44%) was achieved in sample 2-AlMgC-RH14.4 as a result of replacing liquid glass and lignosulfonate with organic condensate from rice husk pyrolysis in the amount of 10.5% and chamotte with rice husk in the amount of 14.4% (Table 5).

**Figure 10:**
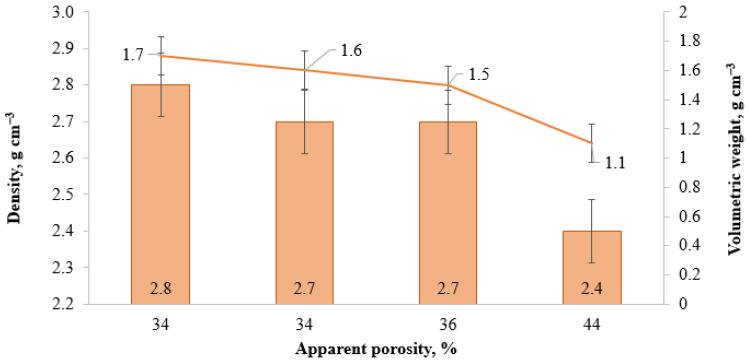
Effect of high-temperature material porosity on their density and volumetric weight.

The density values of the examined samples are presented in Table 5. The control sample (2-AlMgC), containing 2 wt.% of lignosulfonate, exhibited the highest density value of 2.8 g·cm^−3^. Progressive incorporation of organic components into the initial mixtures resulted in a systematic decrease in the density of the resultant high-temperature materials following sintering. The density dropped to 2.7 g·cm^−3^ with the introduction of 2 wt.% of OC-RH (2-AlMgC-SS8.5 sample) and 2 wt.% of OC-RH with 2.4 wt.% of RH (2-AlMgC-RH2.4 sample). In the 2-AlMgC-RH14.4 sample, containing 10.5 wt.% of OC-RH with 14.4 wt.% of RH, the density dropped to 2.4 g·cm^−3^ (Table 5). An analogous trend was observed for volumetric weight. The maximum volumetric weight value (1.7 g·cm^−3^) was, as anticipated, obtained for the control sample 2-AlMgC. With the increase in organic additive content in the initial mixture, the volumetric weight of the sintered samples decreased, attaining a minimum value of 1.1 g·cm^−3^ for experimental sample 2-AlMgC-RH14.4 (Table 5). A similar dependence was observed in [7]; with the introduction of 5 to 20 wt.% of rice husk, the bulk density dropped from 1.5 to 1.23 g·cm^−3^, while in the sample without RH, it was at the level of 1.86 g·cm^−3^. Figure 10 illustrates the correlation between density and volumetric weight of the high-temperature materials as a function of their apparent porosity. As the apparent porosity of the high-temperature materials increased from 34% to 44% (Figure 10), both density (from 2.8 to 2.4 g·cm^−3^) and volumetric weight (from 1.7 to 1.1 g·cm^−3^) decreased correspondingly. Consequently, sample 2-AlMgC-RH14.4 demonstrated the lowest density (2.4 g·cm^−3^) and volumetric weight (1.1 g·cm^−3^) values, corresponding to the highest apparent porosity (44%).

**Figure 11:**
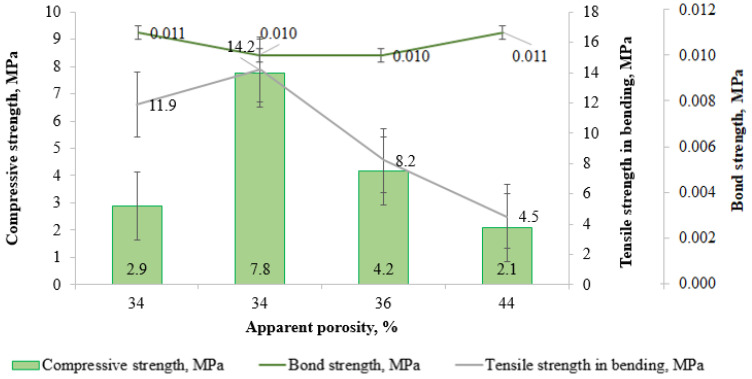
Effect of high-temperature material porosity on strength.

The investigation into the effect of organic substance content and apparent porosity on the mechanical performance (compressive strength and bond strength) of the high-temperature materials revealed no statistically significant correlation between these parameters (Table 5). The highest compressive strength value (7.8 MPa) was exhibited by sample 2-AlMgC-SS8.5. The value of this indicator for sample 2-AlMgC-RH2.4 (4.2 MPa) was almost 2 times lower. The compressive strength of samples 2-AlMgC (2.9 MPa) and 2-AlMgC-RH14.4 (2.1 MPa) is significantly lower than that of the two previous samples, although these samples differ insignificantly from each other in this indicator. The quantity and quality of the binder component likely influence the compressive strength. The combined influence of the filler and the binder on the properties of refractories is also noted in [25]. In the preparation of samples 2-AlMgC-SS8.5 and 2-AlMgC-RH2.4, a sodium silicate solution from rice husk was used as a binder, in contrast to sample 2-AlMgC, which contained commercial liquid glass, and 2-AlMgC-RH14.4, which did not contain a silicate binder at all (Table 4). At equal amounts of the sodium silicate solution from rice husk in the compositions of samples 2-AlMgC-SS8.5 and 2-AlMgC-RH2.4, the latter is naturally characterized by lower compressive strength due to higher apparent porosity (Figure 11), which is caused by the addition of rice husk.

The surface bond strength was determined to assess the possibility of using the tested mixtures as concrete. All samples, as can be seen in Table 5, have practically the same value (0.01 MPa) for this indicator. The influence of composition and apparent porosity on bond strength, unlike compressive strength, turned out to be insignificant (Figure 11). In fact, samples 2-AlMgC, 2-AlMgC-SS8.5, and 2-AlMgC-Rh2.4 have practically the same porosity. The high level of adhesion of sample 2-AlMgC-RH14.4 when determining the bond strength can be explained by the high surface roughness due to the presence of cavities formed as a result of the thermal destruction of organic ingredients.

During the investigations, the obtained samples were also tested for their ability to withstand bending loads, which cause both tensile and compressive stresses in the material (Figure S2e). The maximum tensile strength in bending (14.2 MPa) was exhibited by sample 2-AlMgC-SS8.5, which also demonstrated the highest compressive strength (7.8 MPa). Subsequently, the samples are ranked in descending order of tensile strength in bending as follows: 2-AlMgC (11.9 MPa) > 2-AlMgC-RH2.4 (8.2 MPa) > 2-AlMgC-RH14.4 (4.5 MPa). As can be seen from Table 4 and Table 5 and Figure 11, the mechanical properties of the tested samples are determined using a complex of parameters, including composition and physical properties, and are not only a function of the contents of organic additives or consistent with their porosity indicators.

#### 3.4.2. Thermal Properties

**Figure 12:**
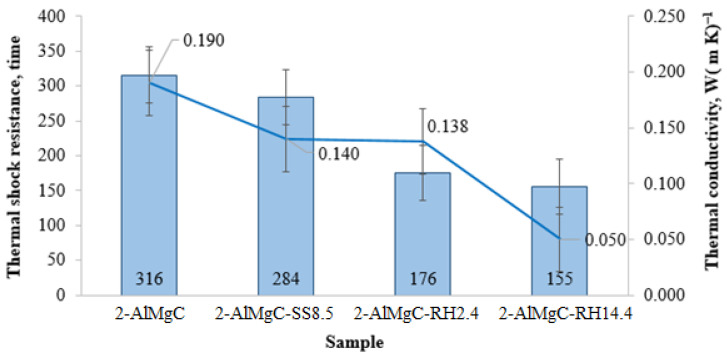
Thermal properties of high-temperature materials.

The thermal properties of the high-temperature materials were evaluated by determining the thermal shock resistance (number of thermal cycles), thermal conductivity (W (m·K)^−1^), and deformation temperature under load (°C). The obtained results are presented in Figure 11. As can be seen from the presented data, the maximum thermal shock resistance (316 thermal cycles) is exhibited by the control sample 2-AlMgC. The replacement of lignosulfonate and liquid glass with rice husk processing products (2-AlMgC-SS8.5 sample) leads to a decrease in the thermal shock resistance indicator to 284 thermal cycles (Figure 12). The introduction of rice husk instead of chamotte contributes to a further decrease in this indicator to 176 (2-AlMgC-RH2.4 sample) and 155 (2-AlMgC-RH14.4 sample) thermal cycles (Figure 12). It should be noted that despite the decrease in thermal shock resistance with the introduction of new materials into the compositions, all samples showed high thermal stability.

An analogous trend is observed in the characteristics of the change in thermal conductivity. The highest thermal conductivity value (0.190 W (m·K)^−1^) was shown by the control sample 2-AlMgC, which has the lowest porosity and the highest density. In contrast, the lowest thermal conductivity value (0.05 W (m·K)^−1^) corresponds to the most porous sample 2-AlMgC-RH14.4. A similar pattern in the change in thermal properties was also observed when using wood sawdust as a burnout additive [6,9].

It should be noted that the value of thermal shock resistance decreased by an average of three times during selective testing of samples of the same composition but of a larger size (50 × 60 mm). This is in good agreement with the known thesis [5] that the value of thermal shock resistance depends on the size of the tested sample and decreases with its increase. Thus, the new synthesized materials, possessing high thermal shock resistance and low thermal conductivity, are promising for use in industry to prevent heat losses.

**Figure 13:**
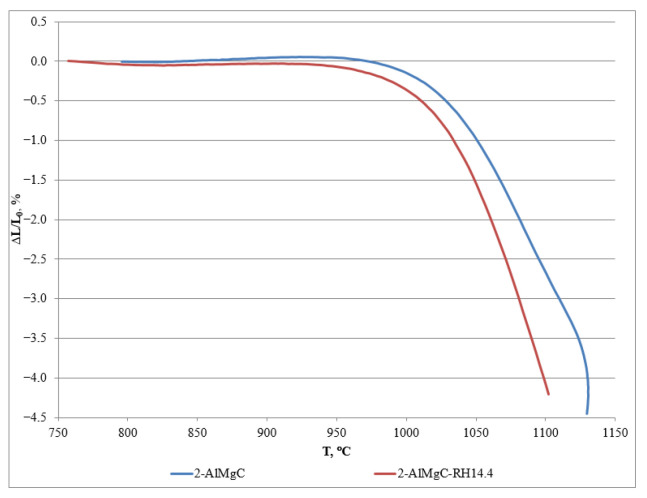
Deformation temperature under load of high-temperature materials.

The sample with the maximum rice husk content (2-AlMgC-RH14.4) was tested for the determination of the deformation temperature under load. The tests involved a comparison with the control sample (2-AlMgC). The deformation temperature of the experimental sample 2-AlMgC-RH14.4 under a load of 0.05 N mm^−2^ was 1100 °C. This value is slightly lower than that of the control sample 2-AlMgC (1120 °C, Figure 13 and Table 6). Consequently, the experimental sample 2-AlMgS-RH14.4 can be used in systems under load up to 1000–1100 °C.

### 3.5. Comparative Analysis of the Properties of Porous High-Temperature Materials

Summarizing the performed investigations, it was of interest to conduct a comparative assessment of the properties of the obtained high-temperature materials with the characteristics of porous refractories reported in the literature. Table 7 presents the compositions and labeling of porous refractories described in the literature. The known high-temperature materials were synthesized using predominantly unground and ground rice husk ash (RHA, S) [6,9,24,25]; rice husk silica (RHS) [22,23]; and rice husk (RH) alone [7]. The studies were performed by replacing part of the diatomite (DE) [6]; diatomaceous silica (DS) [9]; refractory clay (A; Ghanaian red anthill clay, RAC) [7,17]; and kaolin (KC) [22,23]. Mixtures were formulated employing various additives, including sawdust (SD; wood sawdust, WS) [6,9]; wollastonite microfibers (W) [24]; steel fibers (SF) [23]; polysaccharides (PS1), polyvinyl alcohol (PVA), and bentonite (B) [9]; and sodium hexametaphosphate (SHMP) and a sol derived from rice husk ash (Sol) [25]. The refractories were obtained via extrusion (Ex) and pressing (Pr) [9].

It is evident from Table 7 that rice husk ash is utilized as a source of silicon dioxide. The ash content in the refractories varies from 5 to 90%. The most prevalent quantity of rice husk ash incorporated into the refractory compositions ranges from 5 to 20%. As burnout additives, wood sawdust and rice husk were tested in amounts of 5–35 and 5–20 wt.%, respectively. Table 8 presents the physical and mechanical and thermal properties of known porous refractories, prepared according to the compositions from Table 7. For comparison, Table 8 also presents the characteristics of the porous high-temperature materials obtained in the present study.

Analysis of the data presented in Table 7 and Table 8 reveals a consistent trend regarding the influence of biomass additives, aqueous solutions of organic and inorganic compounds, and water content on the physicomechanical and, consequently, thermal properties of refractories. Progressive increases in the quantity of these additives within the mixture result in enhanced porosity and reduced density of the final refractory materials. The following correlation between these parameters was established: refractories exhibiting apparent density or volumetric weight values up to 1 g·cm^−3^ demonstrate porosity exceeding 50% [6,9,25]. The apparent density (volumetric weight) range of 1 to 2 g·cm^−3^ corresponds to porosity values between 13 and 44% ([7,24], present study). When apparent density (volumetric weight) exceeds 2 g·cm^−3^, porosity varies from 13 to 0.4% [22,23]. The porosity parameter significantly influences the mechanical and thermal properties of refractory materials. The observed trends in these property variations are consistent with established theoretical principles governing the relationships among physicomechanical and thermal characteristics. With the increase in porosity, the strength and thermal conductivity of refractories decrease. The thermal shock resistance indicator behaves differently. With an increase in porosity over a wide range (>70%), the thermal shock resistance decreases [6]. In the region of lower porosity values (up to 50%), opposite trends are observed in the change in this indicator [7]. This is consistent with the conclusions of [9], in which the importance of, for example, the orderliness of the refractory’s crystal structure in addition to porosity was shown. Undoubtedly, these are important, but not the only, indicators that determine the performance properties of refractory materials.

In reference [25], a sol from rice husk ash obtained using an analogous method to produce the sodium silicate solution (SS-RH) was used as a binder. Based on this and taking into account the similar conditions for obtaining refractories, it is possible to compare the samples 2-AlMgC-SS8.5 and 2-AlMgC-RH2.4 with sample s-3, 1000 °C [25]. The porosity of the latter (67%) is two times higher than the porosity of our samples 2-AlMgC-SS8.5 (34%) and 2-AlMgC-RH2.4 (36%). The thermal conductivity and compressive strength, however, have almost comparable values (0.132, 0.14, and 0.138 W (m·K)^−1^ and 5.0, 7.8, and 4.2 MPa, respectively). Obviously, this is due to the large consumption of sol compared to SS-RH. This fact, in combination with the results of reference [25], generally reinforces the thesis about the positive influence of SS-RH on the strength of refractories and, consequently, other interrelated properties.

It is also appropriate to compare the characteristics of the experimental sample 2-AlMgC-RH14.4 with the refractory material Anthill-RH15%, obtained based on refractory clay with a 15% replacement by rice husk [7]. As can be seen from Table 8, sample 2-AlMgC-RH14.4 has a higher apparent porosity (44% versus 37%) and a lower thermal conductivity (0.05 W (m·K)^−1^ versus 0.3 W (m·K)^−1^). A significant advantage of 2-AlMgC-RH14.4 is that it is characterized by higher indicators of compressive strength (2.1 MPa versus 1.5 MPa) and thermal shock resistance (155 thermal cycles versus 18). The results of this comparison indicate that the created sample 2-AlMgC-RH14.4, as a porous refractory material, has improved characteristics.

## 4. Conclusions

New high-temperature materials were formulated with the use of rice production waste and its processing products. The basis for their production was the composition of a refractory widely used in industry. The ingredients for its production include refractory clay, chamotte, liquid glass, and lignosulfonate. The mixture contains magnesium sulfate and aluminum. The introduction of these components ensures the possibility of obtaining the refractory via SHS technology. Rice husk was used instead of chamotte in the experimental compositions of refractories. The products of its thermal processing (organic condensate and silicate solution) were used as binders instead of lignosulfonate and liquid glass.

The X-ray diffraction analysis method showed that quartz, tridymite, hematite, mullite, and magnesium aluminosilicate are present in the composition of the formed and prepared firing control and experimental refractories. The formation of the cristobalite phase is observed after firing in the control sample and experimental samples containing new binders and an insignificant amount of rice husk (2.4 wt.%). Only the quartz phase was recorded in the experimental sample with a content of 14.4 wt.% of rice husk and not containing chamotte. After the destruction of the refractories by means of repeated heating at 850 °C and cooling in water at 15 °C, the crystalline phases identified in each of them after firing are preserved in the control and experimental refractories. Along with this, phases of gypsum and kaolinite appear in all samples as a result of the destruction of the clay component, regardless of the use of new raw material resources.

The thermal analysis method established that the main exothermic reaction of self-propagating high-temperature synthesis (exothermic effect at 802 °C) in the aluminum–magnesium sulfate-refractory clay–chamotte system proceeds with the formation of a mineral with an olivine structure (forsterite). When rice husk is introduced into the refractory composition instead of chamotte, this process shifts to higher temperatures. The reaction proceeds with a heat release at 932 °C without changing the mass of the sample as a result of the internal interaction of the components. The microstructure of the experimental refractory, despite its similarity to the control sample (the presence of multiple caverns, the contents of particles of similar sizes from less than 1 μm to more than 100 μm, and the presence of pores), is more friable according to SEM data. This is because the porous formations in the sample with RH are permeated by characteristic interweavings of microfibrils for pyrolyzed rice husk.

During the study of the physicomechanical and thermal properties of the obtained high-temperature materials, it was found that replacing liquid glass with a silicate solution from rice husk contributes to an increase in the compressive strength of the resultant high-temperature materials (7.8 MPa versus 2.9 MPa for the control sample). The use of organic condensate from rice husk pyrolysis as a binder instead of both lignosulfonate and liquid glass is justified. In combination with rice husk (when it is introduced instead of chamotte in the amount of 14.4 wt.%), it promotes the development of apparent porosity (44% relative to 34% in the control sample) with a comparable (2.1 MPa) compressive strength to the control sample (2.9 MPa). The resulting lightweight (volumetric weight 1.1 g·cm^−3^ compared to 1.7 g·cm^−3^ of the control sample) porous refractory is characterized by improved thermal properties (thermal shock resistance—155 thermal cycles, thermal conductivity—0.05 W (m·K)^−1^) compared to refractories known in the literature containing rice husk.

It has been demonstrated in the present study that rice husk and its associated processing products (silicate solution and organic condensate) constitute advantageous precursor materials for the fabrication of porous thermal insulation materials from ecological, economic, and engineering perspectives. New lightweight thermal insulating refractories can be used as an inner lining and an insulation layer. Despite the fact that the goal set in this study has been achieved, it seems necessary to conduct further research. Our subsequent research will be aimed at improving the strength characteristics of the created refractories and studying their behavior in aggressive environments.

## Figures and Tables

**Table 1:** Chemical composition (wt.%) of RH, SCC, and RCC.

Sample	SiO_2_	Al_2_O_3_	P	Ca	Mg	TiO_2_	Mn	Fe	Cu	Pb	Zn	K	Na
RH	20.4	N.D.	0.14	1.86	0.42	0.33	0.034	0.68	0.005	0.006	0.006	0.091	0.052
SCC	36.8	N.D.	0.10	0.94	0.21	0.08	0.029	0.33	0.0005	0.0085	0.010	0.094	0.018

**Table 2:** Elemental composition (wt.%) of RH and SCC.

Sample	C	N	H	S
RH	41.59	1.05	4.32	0.17
SCC	49.02	0.30	0.21	0.44

**Table 3:** XRF analysis of the RCC sample.

Element	Content, wt.%
Si	18.36
P	0.047
S	0.046
K	0.324
Ca	0.285
Ti	0.595
V	0.016
Cr	0.015
Fe	2.08
Zn	0.002
Sr	0.004
Zr	0.036
Nb	0.003
Ba	0.013
Pb	0.002
Bi	0.002
Al	5.23
Balance	72.95

**Table 4:** Composition of high-temperature material mixes (g).

Ingredient	Mix Abbreviation					
	AlMgC	AlMgC-SS8.5	AlMgC-RH2.4	AlMgC-RH14.4	C	C-RH14.4
MgSO_4_·7H_2_SO_4 (tech.)_	4.5	4.5	4.5	5	0	0
Al powder, PAP-1	2	2	2	3	0	0
Refractory clay	59	59	59	67.1	59	67.1
Liquid glass Na_2_SiO_3 (liq.)_	8.5	0	0	0	8.5	0
SS-RH	0	8.5	8.5	0	0	0
Lignosulfonate	2	0	0	0	2	0
OC-RH	0	2	2	10.5	0	10.5
Chamotte	24	24	21.6	0	24	0
RH	0	0	2.4	14.4	0	14.4
Total	100	100	100	100	93.5	92

**Table 5:** Changes in the physical and mechanical properties of high-temperature materials depending on the contents of organic components.

Sample	Lignosulfonate, wt.%	OC-RH, wt.%	RH, wt.%	Apparent Porosity, %	Density, g·cm^−3^	Volumetric Weight, g·cm^−3^	Compressive Strength, MPa	Bond Strength, MPa	Tensile Strength in Bending, MPa
2-AlMgC	2	0	0	34	2.8	1.7	2.9	0.011	11.9
2-AlMgC-SS8.5	0	2	0	34	2.7	1.6	7.8	0.010	14.2
2-AlMgC-RH2.4	0	2	2.4	36	2.7	1.5	4.2	0.010	8.2
2-AlMgC-RH14.4	0	10.5	14.4	44	2.4	1.1	2.1	0.011	4.5

**Table 6:** Softening onset temperature and temperature of height reduction for high-temperature material samples.

Sample	T_06_, °C	T_4_, °C
2-AlMgC	1030	1120
2-AlMgC-RH14.4	1020	1100

**Table 7:** Composition of known porous refractories.

**Sample**	**Raw Materials**							**References**
	DE, wt.%	RHA, wt.%	SD, wt.%	-	-	-	-	
M0	50.0	50.0	0	-	-	-	-	[6]
M5	47.5	47.5	5.0	-	-	-	-	[6]
M10	45.0	45.0	10.0	-	-	-	-	[6]
M15	42.5	42.5	15.0	-	-	-	-	[6]
M20	40.0	40.0	20.0	-	-	-	-	[6]
M25	37.5	37.5	25.0	-	-	-	-	[6]
M30	35.0	35.0	30.0	-	-	-	-	[6]
M35	32.5	32.5	35.0	-	-	-	-	[6]
	RAC, wt.%	SD, wt.%	RH, wt.%	-	-	-	-	
Control	100	0	0	-	-	-	-	[7]
Anthill-SD5%	95	5	0	-	-	-	-	[7]
Anthill-SD10%	90	10	0	-	-	-	-	[7]
Anthill-SD15%	85	15	0	-	-	-	-	[7]
Anthill-SD20%	80	20	0	-	-	-	-	[7]
Anthill-RH5%	95	0	5	-	-	-	-	[7]
Anthill-RH10%	90	0	10	-	-	-	-	[7]
Anthill-RH15%	85	0	15	-	-	-	-	[7]
Anthill-RH20%	80	0	20	-	-	-	-	[7]
Anthill-SD-RH5%	95	2.5	2.5	-	-	-	-	[7]
Anthill-SD-RH10%	90	5	5	-	-	-	-	[7]
Anthill-SD-RH15%	85	7.5	7.5	-	-	-	-	[7]
Anthill-SD-RH20%	80	10	10	-	-	-	-	[7]
	KC, vol.%	RHS, vol.%	-	-	-	-	-	
KC	100	0	-	-	-	-	-	[22]
KS5	95	5	-	-	-	-	-	[22]
KS10	90	10	-	-	-	-	-	[22]
KS20	80	20	-	-	-	-	-	[22]
	KC, vol.%	RHS, vol.%	SF, vol.%	-	-	-	-	
KC	100	0	0	-	-	-	-	[23]
K20S	80	20	0	-	-	-	-	[23]
K20S3F	77	20	3	-	-	-	-	[23]
K20S6F	74	20	6	-	-	-	-	[23]
K20S9F	71	20	9	-	-	-	-	[23]
	A, wt.%	S, wt.%	W, wt.%	-	-	-	-	
A	100	0	0	-	-	-	-	[24]
AS10	90	10	0	-	-	-	-	[24]
AS10W5	85	10	5	-	-	-	-	[24]
AS10W10	80	10	10	-	-	-	-	[24]
AS10W20	70	10	20	-	-	-	-	[24]
	RHA Unground, >200 μm, wt.%	RHA Ground, <200 μm, wt.%	Sol, Dry Sol, wt.%	SHMP, wt.%	Water added, wt.%	Water from Sol, wt.%	SiO_2_, wt.%	
s-1, green, 80 °C	50	47.3	2.5	0.2	5	5.8	-	[25]
s-2, 900 °C	50	44.8	5	0.2	2	11.6	-	[25]
s-3, 1000 °C	50	42.30	7.5	0.2	-	17.5	-	[25]
s-4, 1100 °C	50	39.80	10	0.2	-	23.3	95	[25]
s-5, 1200 °C	50	37.30	12.5	0.2	-	29.2	-	[25]

**Table 8:** Properties of porous refractories.

Sample	Physical and Mechanical Properties					Thermal Properties		References
	Volumetric Weight, g·cm^−3^	Porosity, %	Bending Strength, MPa	Compressive Strength, MPa	Tensile Strength in Bending, MPa	Thermal Shock Resistance at 1000 °C, Time	Thermal Conductivity, W (m·K)^−1^	
M0	0.66	75	1.78	17.35	-	78	0.1035	[6]
M5	0.61	74	1.75	17.24	-	75	0.1024	[6]
M10	0.57	78	1.72	17.03	-	72	0.1003	[6]
M15	0.53	82	1.72	16.84	-	72	0.0984	[6]
M20	0.48	83	1.70	16.68	-	70	0.0968	[6]
M25	0.44	87	1.68	16.41	-	68	0.0941	[6]
M30	0.41	89	1.66	16.11	-	66	0.0911	[6]
M35	0.37	92	1.61	15.78	-	61	0.0878	[6]
Control	1.86 ^4^	25 ^5^	-	2.80	-	1 ^3^	0.55	[7]
Anthill-SD5%	1.42 ^4^	33 ^5^	-	1.82	-	11 ^3^	0.46	[7]
Anthill-SD10%	1.28 ^4^	39 ^5^	-	1.38	-	16 ^3^	0.40	[7]
Anthill-SD15%	1.14 ^4^	43 ^5^	-	1.1	-	21 ^3^	0.35	[7]
Anthill-SD20%	1.07 ^4^	53 ^5^	-	0.74	-	25 ^3^	0.31	[7]
Anthill-RH5%	1.52 ^4^	23 ^5^	-	2.32	-	8 ^3^	0.40	[7]
Anthill-RH10%	1.36 ^4^	34 ^5^	-	1.80	-	13 ^3^	0.33	[7]
Anthill-RH15%	1.2 ^4^	37 ^5^	-	1.50	-	18 ^3^	0.30	[7]
Anthill-RH20%	1.23 ^4^	40 ^5^	-	1.05	-	20 ^3^	0.23	[7]
Anthill-SD-RH5%	1.5 ^4^	31 ^5^	-	2.09	-	10 ^3^	0.44	[7]
Anthill-SD-RH10%	1.3 ^4^	38 ^5^	-	1.60	-	14 ^3^	0.38	[7]
Anthill-SD-RH15%	1.22 ^4^	41 ^5^	-	1.12	-	19 ^3^	0.32	[7]
Anthill-SD-RH20%	1.15 ^4^	43 ^5^	-	0.9	-	22.5 ^3^	0.25	[7]
KC	2.25 ^2^	3	-	116.93	19.26	0.46 ^3^	-	[22]
KS5	2.25 ^2^	2	-	115.0	18.75	0.47 ^3^	-	[22]
KS10	2.25 ^2^	1	-	129.25	24.13	0.47 ^3^	-	[22]
KS20	2.25 ^2^	0.4	-	140.06	27.98	0.73 ^3^	-	[22]
KC	2.15 ^2^	12	-	-	24.41	1 ^3^	-	[23]
K20S	2.25 ^2^	9	-	-	28.34	1 ^3^	-	[23]
K20S3F	2.35 ^2^	11	-	-	14.00	0.25 ^3^	-	[23]
K20S6F	2.45 ^2^	13	-	-	13.75	0.27 ^3^	-	[23]
K20S9F	2.60 ^2^	13	-	-	13.00	0.36 ^3^	-	[23]
A	1.66 ^1^	13	-	-	-	1	0.1659	[24]
AS10	1.54 ^1^	16	-	-	-	1	0.1623	[24]
AS10W5	1.47 ^1^	19	-	-	-	1	0.1559	[24]
AS10W10	1.59 ^1^	23	-	-	-	1	0.1523	[24]
AS10W20	1.53 ^1^	25	-	-	-	1	0.1446	[24]
s-1, green, 80 °C	-	-	-	-	-	-	-	[25]
s-2, 900 °C	0.73 ^2^	69 ^5^	-	4.1 ^6^	-	-	0.124	[25]
s-3, 1000 °C	0.76 ^2^	67 ^5^	-	5.0 ^6^	-	-	0.132	[25]
s-4, 1100 °C	0.79 ^2^	64 ^5^	-	5.4 ^6^	-	-	0.135 at 30 °C	[25]
s-5, 1200 °C	0.90 ^2^	59 ^5^	-	6.5 ^6^	-	-	0.146	[25]
2–0	1.7/2.8 ^7^	34 ^5^	0.011 ^8^	2.9	11.9	316 ^9^	0.19	Present study
2–4	1.6/2.7 ^7^	34 ^5^	0.010 ^8^	7.8	14.2	284 ^9^	0.14	Present study
2–9a	1.5/2.7 ^7^	36 ^5^	0.010 ^8^	4.2	8.2	176 ^9^	0.138	Present study
2–13 g	1.1/2.4 ^7^	44 ^5^	0.011 ^8^	2.1	4.5	155 ^9^	0.05	Present study

^1^ Bulk density, g·cm^−3^. ^2^ Apparent bulk density, g·cm^−3^. ^3^ At 1200 °C. ^4^ Apparent density, g·cm^−3^. ^5^ Apparent porosity, %. ^6^ Cold compressive strength, MPa. ^7^ True density, g·cm^−3^. ^8^ Bond strength, MPa. ^9^ At 850 °C.

## Data Availability

The original contributions presented in this study are included in the article/Supplementary Materials. Further inquiries can be directed to the corresponding author.

## Supplementary Materials

The following supporting information can be downloaded at: https://www.mdpi.com/article/10.3390/ma18215063/s1, Figure S1: Chromatogram of OC-RH; Figure S2: High-temperature material property testing; Figure S3: SEM-EDS image (example 1) of the sample 1-AlMgC-RH14.4; Figure S4: SEM-EDS image (example 2) of the sample 1-AlMgC-RH14.4; Figure S5: SEM-EDS image (example 1) of the sample 1-AlMgC; Figure S6: SEM-EDS image (example 2) of the sample 1-AlMgC; Table S1: GC-MS analysis of OC-RH.

## Abbreviations

The following abbreviations are used in the manuscript and Supplementary Materials:

A	Refractory clay
A_i_	Bond strength, MPa
AlMgC	Composition of control sample of high-temperature material containing (wt.%): MgSO_4_·7H_2_O—4.5; Al powder—2; refractory clay—59; liquid glass—8.5; lignosulfonate—2; chamotte—24
1-AlMgC	AlMgC sample dried (Series 1)
2-AlMgC	AlMgC sample fired (Series 2)
3-AlMgC	AlMgC sample destroyed during the thermal shock resistance test (Series 3)
AlMgC-RH2.4	Composition of experimental sample of high-temperature material containing (wt.%): MgSO_4_·7H_2_O—4.5; Al powder—2; refractory clay—59; sodium silicate solution from rice husk—8.5; organic condensate from rice husk pyrolysis—2; rice husk—2.4; chamotte—21.6
1-AlMgC-RH2.4	AlMgC-RH2.4 sample dried (Series 1)
2-AlMgC-RH2.4	AlMgC-RH2.4 sample fired (Series 2)
3-AlMgC-RH2.4	AlMgC-RH2.4 sample destroyed during the thermal shock resistance test (Series 3)
AlMgC-RH14.4	Composition of experimental sample of high-temperature material containing (wt.%): MgSO_4_·7H_2_O—5; Al powder—3; refractory clay—67.1; organic condensate from rice husk pyrolysis—10.5; rice husk—14.4
1-AlMgC-RH14.4	AlMgC-RH14.4 sample dried (Series 1)
2-AlMgC-RH14.4	AlMgC-RH14.4 sample fired (Series 2)
3-AlMgC-RH14.4	AlMgC-RH14.4 sample destroyed during the thermal shock resistance test (Series 3)
AlMgC-SS8.5	Composition of experimental sample of high-temperature material containing (wt.%): MgSO_4_·7H_2_O—4.5; Al powder—2; refractory clay—59; silicate solution from rice husk—8.5; organic condensate from rice husk pyrolysis—2; chamotte—24
1-AlMgC-SS8.5	AlMgC-SS8.5 sample dried (Series 1)
2-AlMgC-SS8.5	AlMgC-SS8.5 sample fired (Series 2)
3-AlMgC-SS8.5	AlMgC-SS8.5 sample destroyed during the thermal shock resistance test (Series 3)
B	Bentonite
C	AlMgC sample prepared without MgSO_4_·7H_2_O and Al powder
1-C	C sample dried (Series 1)
C-RH14.4	AlMgC-RH14.4 sample prepared without MgSO_4_·7H_2_O and Al powder
1-C-RH14.4	C-RH14.4 sample dried (Series 1)
DE	Diatomite
DS	Industrial diatomaceous silica
EDS	Energy dispersive spectroscopy
Ex	Extrusion method
GC-MS	Gas chromatography–mass spectrometry
KC	Kaolin
OC-RH	Organic condensate prepared by rice husk pyrolysis
PAP-1	Grade aluminum powder
Pr	Pressing method
PS1	Polysaccharide
PVA	Polyvinyl alcohol
RAC	Red anthill clay
RHA	Rice husk ash
RHS	Rice husk silica
R_tb_	Tensile strength in bending, MPa
S	Rice husk ash
SCC	Silicon carbon composite
SCC-V	SCC sample activated with water steam at 850 °C for 30 min
SCC-VA	SCC-V sample activated by alkali
SD	Sawdust
SF	Steel fibers
SHMP	Sodium hexametaphosphate
Sol	Sol derived from rice husk ash
SRC	Silicon carbide
W	Wollastonite
WS	Waste sediment
σ	Ultimate compressive strength, MPa
λ	Thermal conductivity, W (m·K)^−1^
ρ	True density, g·cm^−3^
