# Supplementary material for: Porous Refractories Synthesized Using Rice Husk and Rice Husk Processing Products
Source: Materials (Basel). 2025 Nov 6;18(21):5063. doi: 10.3390/ma18215063 (PMC12608176; doi:10.3390/ma18215063)
Supplement: Supplementary file 1 [file materials-18-05063-s001.zip › materials-3945976-supplementary.pdf]

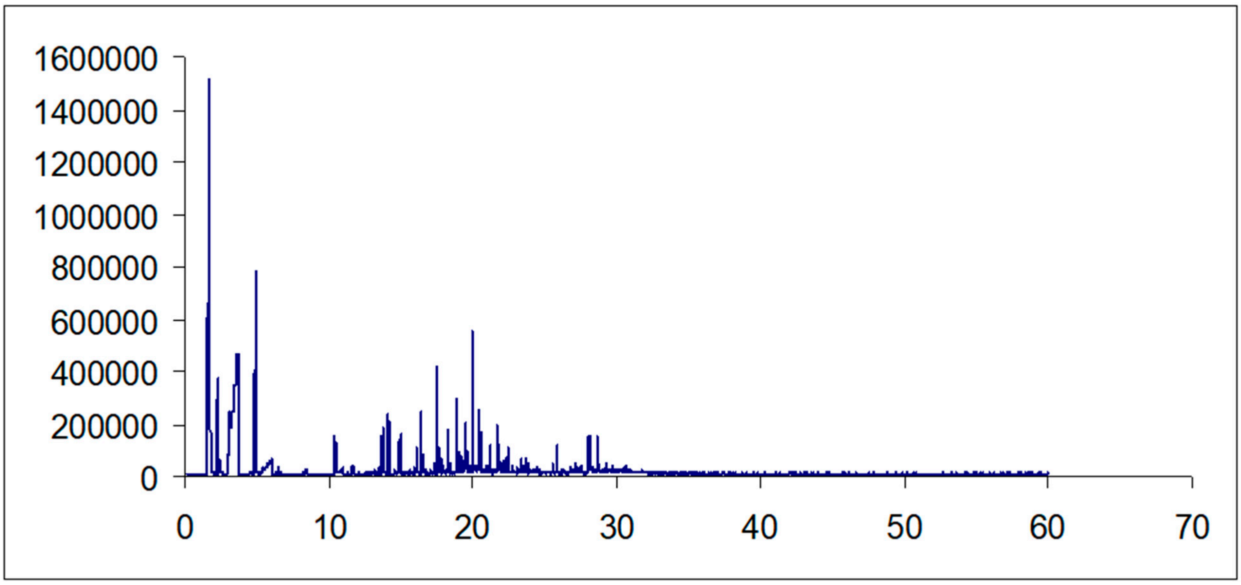

Figure S1. Chromatogram of OC-RH.

Table S1. GC-MS analysis of OC-RH.

RT, min	Compound Name	Mass Fraction*, %
1.73	Water	24,83
2.12	Acetone	1,57
2.30	Formic acid	0,32
2.35	Acetic acid, methyl ester	1,96
2.97	Acetic acid	19,52
4.89	2-Propanone, 1-hydroxy-	6,44
5.62	Propanoic acid	3,12
6.50	2-Butanone, 3-hydroxy-	0,28
8.41	Pyridine	0,31
10.35	1-Hydroxy-2-butanone	2,04
11.64	Cyclopentanone	0,33
12.73	Pyridine, 2-methyl-	0,11
13.68	Furfural	0,93
13.74	2-Cyclopenten-1-one	1,43
14.16	3-Furanmethanol	1,64
14.51	1-Hydroxy-2-pentanone	0,11
14.56	Pyridine, 3-methyl-	0,09
14.91	1,2-Ethanediol, diacetate	0,81
16.05	2-Cyclopenten-1-one, 2-methyl- ...	0,53
16.11	Ethanone, 1-(2-furanyl)-	0,58
16.59	Pyridine, 2,5-dimethyl-	0,10
16.73	2,5-Hexanedione	0,08
17.06	2(5H)-Furanone, 5-methyl-	0,10
17.50	Phenol	2,30
17.70	2-Cyclopenten-1-one, 3-methyl-	0,69
17.99	2(5H)-Furanone, 3-methyl-	0,20
18.30	Pyridine, 3-methoxy-	0,13
18.85	2-Cyclopenten-1-one, 2-hydroxy-...	1,70
19.11	Phenol, 2-methyl-	0,52
19.19	2-Cyclopenten-1-one, 2,3-dimethyl-	0,36

19.32	3,5-dimethyl cyclopentenolone	0,25
19.53	Phenol, 4-methyl-	0,66
19.57	Phenol, 3-methyl-	0,53
19.97	Phenol, 2-methoxy-	2,88
21.17	Phenol, 4-ethyl-	0,56
21.67	1,2-Benzenediol	1,46
21.88	2-methoxy-4-methylphenol	0,67
23.15	1,2-Benzenediol, 3-methyl-	0,32
23.37	Hydroquinone	1,03
23.72	Phenol, 4-ethyl-2-methoxy-	0,33
23.88	1,2-Benzenediol, 4-methyl-	0,51
25.52	1,4-Benzenediol, 2-methyl-	0,39
25.84	Phenol, 2,6-dimethoxy-	0,69
27.06	1,3-Benzenediol, 4-ethyl-	0,21
30.48	Ethanone, 1-(4-hydroxy-3,5-dime...	0,14

* The percentage calculation is performed by the peak area normalization method, the actual concentration may differ (calibration required).

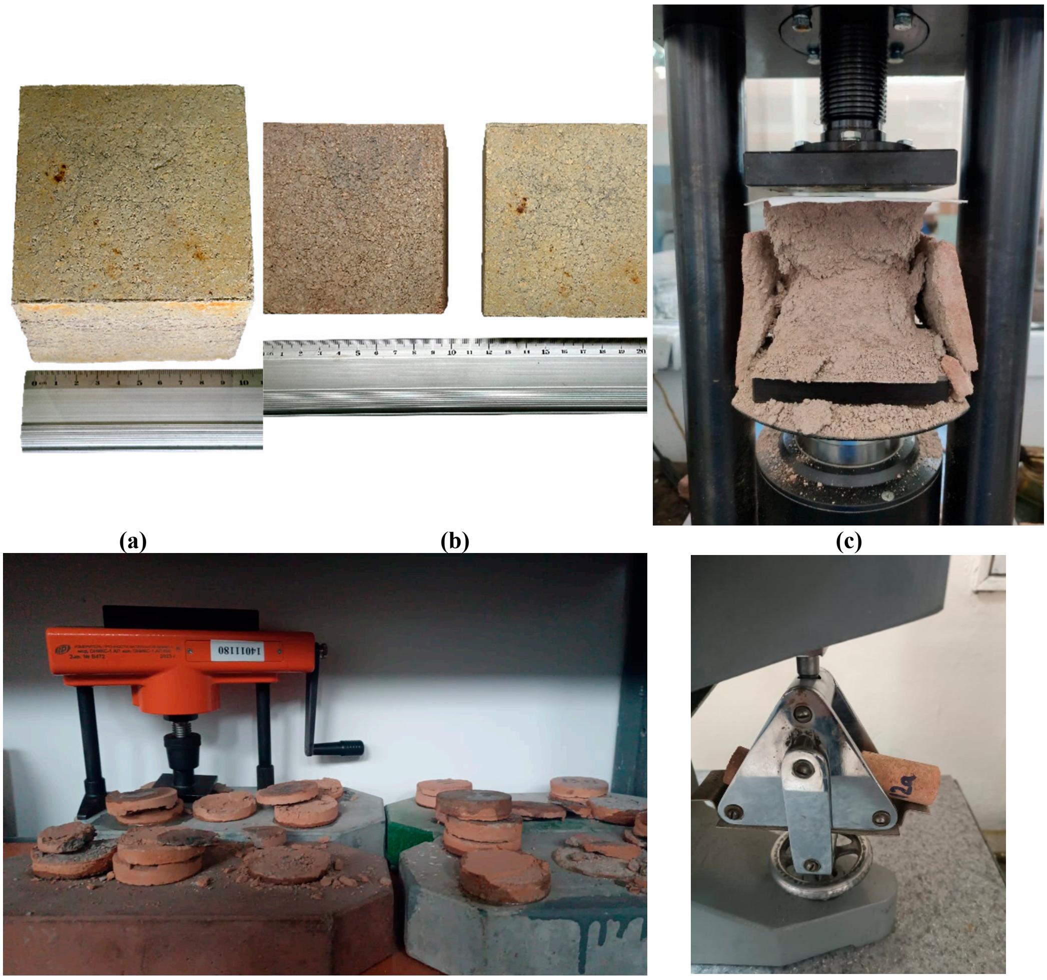

(a) (b) (c) (d) (e)

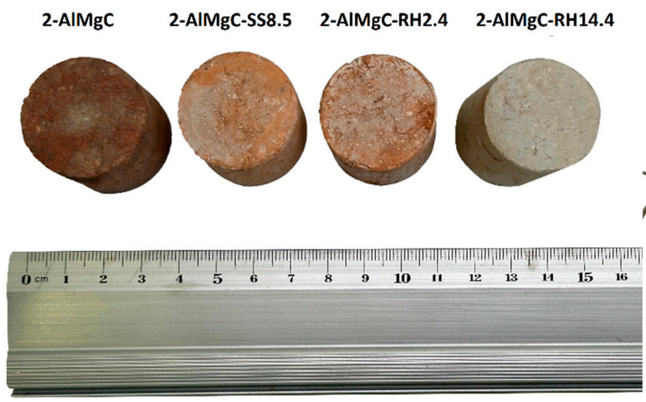

(f)

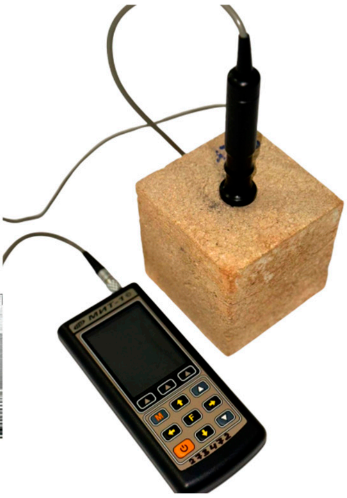

(g)

Figure S2. High-temperature material property testing: **(a)**, **(b)** Cubic samples for the compressive strength determination; **(c)** Compressive strength determination; **(d)** Bond strength determination; **(e)** Tensile strength in bending determination; **(f)** Cylindrical samples for the thermal shock resistance determination; **(g)** Thermal conductivity determination.

001

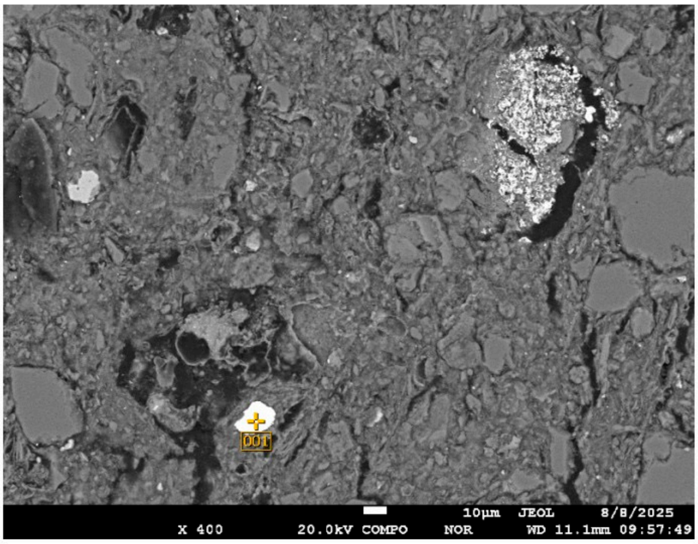

Volt : 20.00 kV
Mag. : x 400
Date : 2025/08/08
Pixel : 1280 x 960

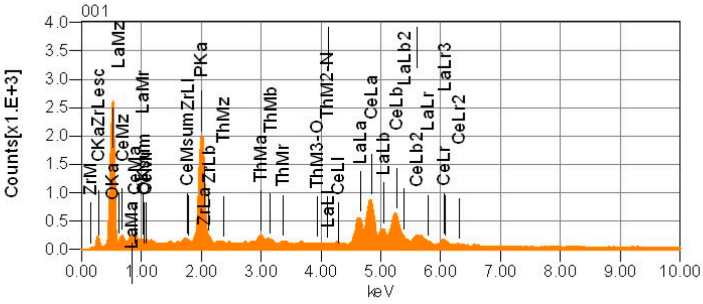

Acquisition Condition
Instrument : 8230
Volt : 20.00 kV
Current : 0.00 nA
Process Time : T3
Live time : 60.00 sec.
Real Time : 69.00 sec.
DeadTime : 13.00 %
Count Rate : 3181.00 CPS

Chemical formula	ms%	mol%	Sigma	Net	K ratio	Line
C	4.58	11.44	0.11	23205	0.0019802	K
O	36.03	67.51	0.13	541952	0.2014578	K
P	10.75	10.41	0.10	621700	0.0981888	K
Zr	4.40	1.45	0.30	153737	0.0283794	L
La	14.73	3.18	0.40	380591	0.1372174	L
Ce	26.10	5.58	0.39	694893	0.2555225	L
Th*	3.41	0.44	0.37	90950	0.0243924	M
Total	100.00	100.00				

JEOL EDS System JEOL

Figure S3. SEM-EDS image (example 1) of the sample 1-AlMgC-RH14.4.

002

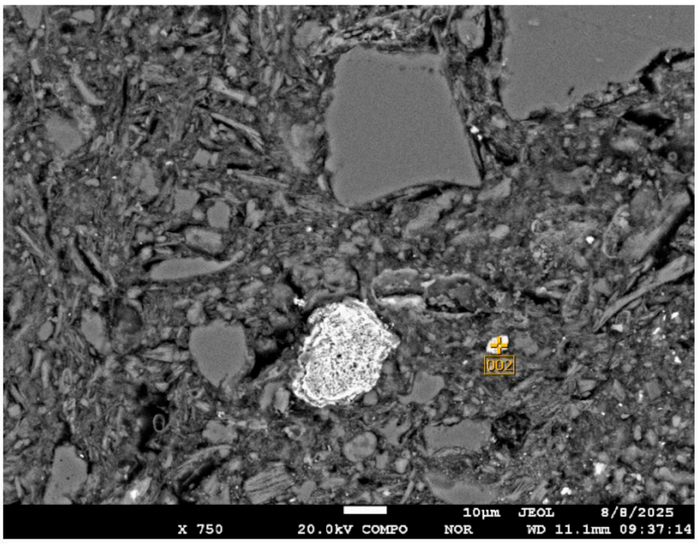

Volt : 20.00 kV
Mag. : x 750
Date : 2025/08/08
Pixel : 1280 x 960

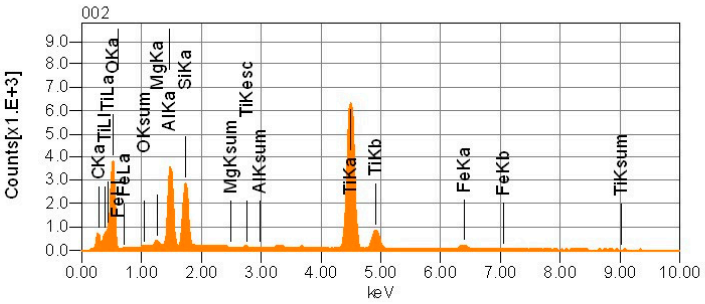

Acquisition Condition
Instrument : 8230
Volt : 20.00 kV
Current : 0.00 nA
Process Time : T3
Live time : 60.00 sec.
Real Time : 70.33 sec.
DeadTime : 15.00 %
Count Rate : 4983.00 CPS

Chemical formula		ms%	mol%	Sigma	Net	K ratio	Line
C	6.72	11.88	0.09	94963	0.0036041		K
O	48.41	64.23	0.29	763336	0.1262008		K
Mg	0.51	0.45	0.08	61372	0.0027328		K
Al	6.47	5.09	0.07	984064	0.0440059		K
Si	5.34	4.04	0.07	853246	0.0417316		K
Ti	30.98	13.73	0.14	3181199	0.3159798		K
Fe	1.57	0.60	0.27	91614	0.0154956		K
Total	100.00	100.00					

Figure S4. SEM-EDS image (example 2) of the sample 1-AlMgC-RH14.4.

001

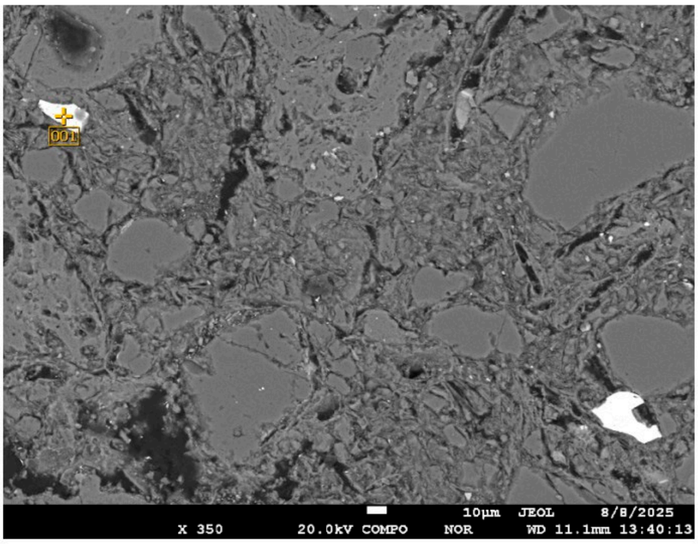

Volt : 20.00 kV
Mag. : x 350
Date : 2025/08/08
Pixel : 1280 x 960

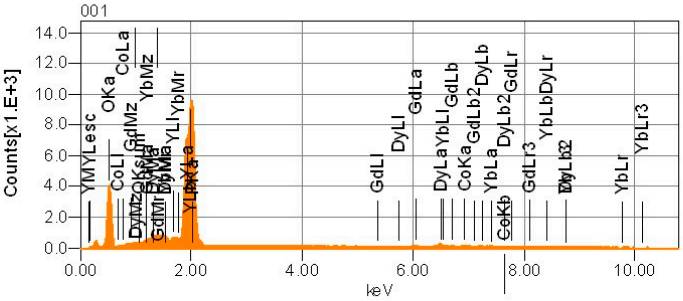

Acquisition Condition
Instrument : 8230
Volt : 20.00 kV
Current : 0.00 nA
Process Time : T3
Live time : 60.00 sec.
Real Time : 71.52 sec.
DeadTime : 15.00 %
Count Rate : 6701.00 CPS

Chemical formula	ms%	mol%	Sigma	Net	K ratio	Line
O	40.42	72.65	0.20	860086	0.1463417	K
P*	15.07	13.99	0.07	2354530	0.1702112	K
Co	1.04	0.51	0.34	51338	0.0106143	K
Y*	35.35	11.43	0.25	3253983	0.2667182	L
Gd*	1.76	0.32	0.63	66288	0.0148096	L
Dy*	4.23	0.75	0.75	137191	0.0355229	L
Yb*	2.13	0.35	0.38	89014	0.0147317	M
Total	100.00	100.00				

JEOL EDS System

Figure S5. SEM-EDS image (example 1) of the sample 1-AlMgC.

002

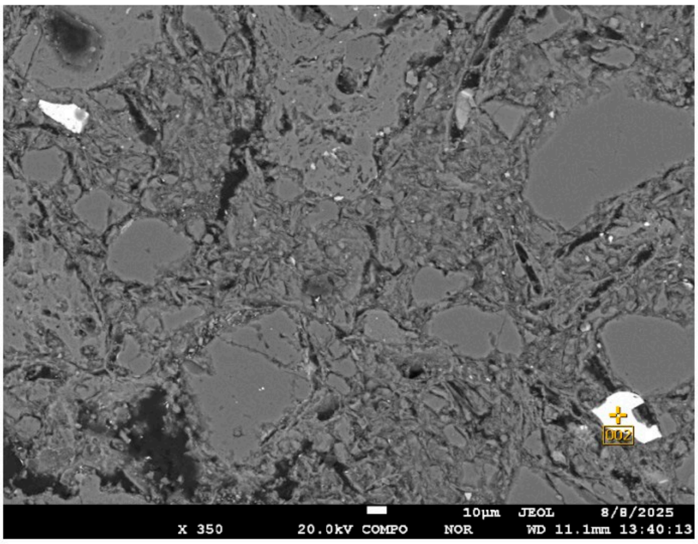

Volt : 20.00 kV
Mag. : x 350
Date : 2025/08/08
Pixel : 1280 x 960

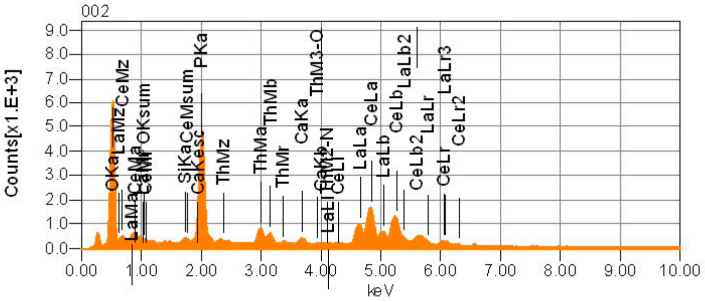

Acquisition Condition
Instrument : 8230
Volt : 20.00 kV
Current : 0.00 nA
Process Time : T3
Live time : 60.00 sec.
Real Time : 71.31 sec.
DeadTime : 16.00 %
Count Rate : 6621.00 CPS

Chemical formula	ms%	mol%	Sigma	Net	K ratio	Line
O	38.98	76.07	0.16	1294876	0.2207536	K
Si	0.33	0.36	0.12	38574	0.0019454	K
P	13.02	13.13	0.11	1554927	0.1126282	K
Ca	0.92	0.72	0.15	110157	0.0096972	K
La	13.12	2.95	0.52	695230	0.1149570	L
Ce	25.43	5.67	0.52	1387786	0.2340396	L
Th*	8.20	1.10	0.47	462535	0.0568920	M
Total	100.00	100.00				

JEOL EDS System

Figure S6. SEM-EDS image (example 2) of the sample 1-AlMgC.
